# Anticipating Greater Impact of the COVID-19 Pandemic on Social Life Is Associated With Reduced Adherence to Disease-Mitigating Guidelines

**DOI:** 10.3389/fpsyg.2021.756549

**Published:** 2022-01-27

**Authors:** Rista C. Plate, Adrianna C. Jenkins

**Affiliations:** Department of Psychology, University of Pennsylvania, Philadelphia, PA, United States

**Keywords:** COVID-19, coronavirus, social behavior, disease prevention, social norms

## Abstract

People regularly make decisions about how often and with whom to interact. During an epidemic of communicable disease, these decisions gain new weight, as individual choices exert more direct influence on collective health and wellbeing. While much attention has been paid to how people’s concerns about the health impact of the COVID-19 pandemic affect their engagement in behaviors that could curb (or accelerate) the spread of the disease, less is understood about how people’s concerns about the pandemic’s impact on their social lives affect these outcomes. Across three studies (total *N* = 654), we find that individuals’ estimates of the pandemic’s social (vs. health) impact are associated with an unwillingness to curtail social interaction and follow other Centers for Disease Control guidelines as the pandemic spreads. First, these associations are present in self-report data of participants’ own behaviors and behavior across hypothetical scenarios; second, participants’ estimates of the pandemic’s impact on social life in their location of residence are associated with movement data collected unobtrusively from mobile phones in those locations. We suggest that perceptions of social impact could be a potential mechanism underlying, and therefore potential intervention target for addressing, disease-preventing behavior during a pandemic.

## Introduction

People often face tradeoffs between their own immediate interests and the collective interests of a group. In March 2020, the World Health Organization declared a global pandemic as a result of the worldwide spread of a new coronavirus (SARS-CoV-2), which causes the respiratory disease COVID-19. The declaration of a pandemic brought with it a collection of guidelines aimed at curtailing the spread of the disease and that imposed limitations on people’s activities in daily life. These guidelines were met with widely varying levels of compliance across individuals (Marcus, 2020; Wiest, 2020), in turn affecting the spread of the disease. Indeed, it is estimated that adhering to one specific type of guideline—strict reduction in one’s direct interactions with others (i.e., “social distancing”)—could have saved tens of thousands of lives in the 1st year of the pandemic (IHME Covid-19 Forecasting Team, 2021).

These figures point to the importance of understanding the factors that give rise to compliance with guidelines aimed at mitigating disease spread. In particular, although individual differences in demographics (e.g., age, race, gender, political orientation; Alsan et al., 2020; Rothgerber et al., 2020), personal beliefs (e.g., political beliefs, Gollwitzer et al., 2020; trust in science, Plohl and Musil, 2021), and personality (e.g., agreeableness, conscientiousness; Blagov, 2020; Bogg and Milad, 2020) have been shown to play important roles in people’s adherence to recommended guidelines, the relative stability of these attributes makes them unlikely to be viable targets of intervention. By contrast, people’s *perceptions* of the potential impact of the pandemic on various aspects of human life are potentially more malleable. Additionally, perceptions may change over time as the overall picture of the pandemic continues to evolve (e.g., in terms of case prevalence, information regarding transmission, treatment options).

Although perceptions of the health consequences of the pandemic have been well studied, people’s perceptions of the impact of the pandemic on social life are less well understood, despite the fact that there have been dramatic changes to daily social life and social interactions (e.g., school closures, masking in indoor spaces). Here, we investigate the possibility that individual differences in the perceived consequences of the pandemic for specific aspects of human life are associated with disease-preventing behaviors in specific ways. In particular, we test the possibility that the anticipated impact of the pandemic on social life, over and above its anticipated health consequences, relates to people’s (un)willingness to curtail social behavior in the interest of mitigating disease spread.

Behavioral interventions have proven effective for containment of COVID-19 and have a robust potential to prevent illness and death (Anderson et al., 2020). As a result, there is a need to understand what factors might influence whether individuals elect to engage in behaviors that health experts recommend (Bavel et al., 2020). Initial evidence points to the possibility that people’s beliefs and perceptions may play an important role in their compliance with health guidelines (Xie et al., 2020; Zajenkowski et al., 2020). For example, increasing awareness of risk of transmission was associated with an increase in adherence to recommended behaviors (e.g., handwashing, social distancing; Wise et al., 2020). Additionally, those who had higher optimism regarding the pandemic were more likely to adhere to social distancing (Sheetal et al., 2020). Research has focused almost exclusively on people’s perceptions of the potential health consequences of the pandemic. Yet health is not the only area of impact. There have been extreme changes to our social habits, behaviors, and relationships. People typically turn to their social connections and relationships during times of hardship to promote wellbeing (Jetten et al., 2017; Williams et al., 2018) and social distance (which can be accompanied by social disconnection) runs counter to these human tendencies, resulting in serious personal health and mental health concerns (Baumeister and Leary, 1995; Hawkley and Cacioppo, 2010; Chen et al., 2020).

While perceptions related to the health impact of the pandemic have been associated with engagement in preventative behaviors (Sheetal et al., 2020; Wise et al., 2020), there are reasons to think that perceptions related to the social impact of the pandemic could have different effects. On the one hand, perceived social impact could increase behavior that is likely to spread, rather than curtail the spread of, the virus. In one study, those with less social stability—whose social relationships were perhaps more vulnerable to the effects of distancing recommendations (e.g., reduced opportunities to pursue new social connections because of fewer occasions to interact with strangers)—were less likely to adhere to distancing guidelines (Corpuz et al., 2020). A similar idea has been examined at the global level (Salvador et al., 2020). Countries higher in relational mobility, defined as the degree of relationship change in the society (Yuki and Schug, 2020), had a faster growth rate of cases of COVID-19 compared to countries lower in relational mobility. Although correlational, one possibility is that individuals in these areas perceive a more significant threat of the pandemic to social life and therefore opt to preserve social engagement instead of social distancing. On the other hand, elevated estimates of the social impact of the pandemic could increase disease-mitigating behavior. For example, greater perceived threat to social connection might lead people to try to minimize the duration of the threat (e.g., by adhering more strictly to guidelines) if they feel empowered to address it (Pfattheicher et al., 2020).

Here, we assessed perceptions of the health and social impacts of the pandemic in separate samples across two time points: 3 weeks after the pandemic declaration (April 2020) and 13 weeks after the pandemic declaration (June 2020). Between April and June 2020, much changed in terms of pandemic, including the cumulative number of cases and deaths, the current number of cases, and the knowledge available regarding transmission, effective preventative measures, and the course of the virus (CDC COVID-19 Response Team, 2020; Batova, 2021). Additionally, by June there had been more time to experience the social impact of the pandemic as a result of sustained school closures and the passing of holidays that people otherwise often spend with family and friends (e.g., Easter, Passover). By measuring perceptions of the health and social impact of the pandemic across both people and time, we can leverage individual and time-related differences to get traction on the question of how those perceptions might relate to behavior across the changing landscape of the pandemic.

In Study 1, we first examined whether there are differences in the perceived severity of the pandemic’s impact on health and social domains and whether the perceived severity of the pandemic’s impact on health and social domains was different in April and June. Next, we tested to what extent people’s perceptions of impact are associated with their likelihood of engaging in disease-preventing behaviors. We were particularly interested in whether perceived social impacts would be associated with more or less engagement with disease-preventing behaviors, including social distancing. Finally, we examined the relationship between ratings of perceived impact and changes in the extent of people’s physical movement through space, as indexed by state-level movement change using Google movement data. We included the Google movement data as an out-of-sample behavioral measure of the extent to which individuals followed social distancing recommendations. State-level movement provides a proximal and objective measure of social activity.

In Study 2, we assessed whether people’s perceptions of the impact of the pandemic, and behavioral intentions, could be influenced by explicit messages about the pandemic’s social or health impacts. In Study 2, we also asked participants to estimate rates of disease prevalence that would influence their movement to the specific locations indexed by the Google movement data in order to tie the state-level movement outcomes to individual-level movement predictions. Across both Study 1 and Study 2, we predicted that perceptions of social impact of the pandemic would be associated with behavior over and above perceived health impacts. In doing so, we aimed to identify a potential mechanism for understanding whether, and why, individuals might continue to engage with others when doing so poses a health risk. Finally, in Study 3, we explored reasons for an association between perceptions of the social impact of the pandemic and behavior. Specifically, we asked to what extent behaviors associated with curbing social interactions (e.g., staying away from family and friends) were rated as more difficult, less controllable, or less effective than other disease-mitigating actions (e.g., wearing a mask). Better understanding such factors may help guide public health communication and recommendations.

## Study 1

Study 1 sought to characterize the anticipated impact of the pandemic on health and social domains across time and to investigate how these expectations relate to people’s disease-mitigating behaviors. In order to assess whether perceived social impacts could relate to behavior over and above health influences, we first needed to compare perceptions of health and social impacts to each other and across time. Therefore, we examined ratings of perceived health impacts in two samples, one collected 3 weeks after the pandemic declaration and one collected 13 weeks after the pandemic declaration. Then, we examined whether the perceived social impact of the pandemic was associated with behavior, and whether the direction of association was consistent with disease-mitigating behaviors. Finally, we examined these associations using out-of-sample data to better understand how individual ratings relate to larger patterns of population movement.

### Method

For all studies, we report how we determined our sample size, all data exclusions, and all manipulations. Because the studies reported here were part of a larger data collection effort related to the COVID-19 pandemic, measures were collected outside the scope of the current hypotheses. All measures collected in the full study are reported and available on: https://osf.io/48yxk/

#### Participants

Participants were two groups of adults (total *n* = 188) recruited via Amazon’s Mechanical Turk approximately 3 weeks or approximately 13 weeks after the World Health Organization declared the COVID-19 outbreak a global pandemic. Ninety-seven participants completed the study on April 3, 2020 (45 female, 52 male; *age range* = 22-73 years, *M*_*age*_ = 38.52, *SD*_*age*_ = 12.53) and an independent set of 91 participants completed the study on June 15, 2020 (39 female, 52 male; *age range* = 19-69 years, *M*_*age*_ = 39.56, *SD*_*age*_ = 11.44; with the exception that seven participants had also completed the survey in April). We restricted participation to MTurk workers with HIT acceptance rates > 97% who were located in the United States. Sixty-four additional participants were excluded because of bot-like responses (e.g., material directly pasted from websites, identical responses across multiple participants), using a restricted number of response options, or currently residing outside of the United States. Participants provided informed consent and received $1.25 for completing the task. The study was approved by the Institutional Review Board.

#### Materials and Procedure

Participants completed a questionnaire assessing their individual perceptions of the expected impact of the COVID-19 pandemic (See Supplementary Table 1 for measures across studies). First, participants provided overall ratings of the degree to which they expected consequences be severe (i) in the next 3 months and (ii) in 1 year, separately for “health-related consequences” and “social-related consequences,” on a 7-point scale from 1 = not at all severe to 7 = extremely severe. We refer to these variables as “perceived health impact” and “perceived social impact.”

Next, to assess disease-preventing and -promoting behaviors, participants rated how likely they were to engage in each of 26 activities that spanned daily living activities, including activities in which one would interact with other people (e.g., patron a local business, get within 6 feet of an elderly individual) within the next week (both sets of participants) and in the following month (April participants only) on a 5-point scale from 1 = not at all likely to 5 = extremely likely. Finally, participants provided demographic information, including the state in which they were currently residing and the number of COVID-19 cases in their city. We report findings investigating relationships moderated by local prevalence of cases in Supplementary Materials (“Effects of Perceived Impact on Behavior Vary by Local Prevalence”).

To identify core dimensions underlying the 26 behaviors, we conducted an exploratory factor analysis. The parallel analysis and scree plot recommended three factors. The three-factor solution using a varimax rotation accounted for 51% of the variance (see Supplementary Table 2 for factor loadings; while small, the amount of variance explained is within the common bounds for social science research, Williams et al., 2010). The first factor, which explained 29% of the variance, primarily contained variables related to directly interacting with others (e.g., travel, interacting with neighbors). We refer to this factor as the “intentional social interactions” factor because it signals intentional social interactions (as opposed to indirect or incidental social interactions such as being in close proximity to others at the grocery store). Other variables related to interacting with others loaded on the second factor, which explained 12% of the variance. This “everyday social interactions” factor included variables related to day-to-day interactions (e.g., with household members; reporting of the everyday social interactions factor is included in the Supplementary Materials). The third factor, which explained 10% of the variance, contained behaviors related to recommendations for limiting the spread of the virus that were not focused on directly interacting with others, including washing one’s hands and following recommended guidelines generally. We will refer to this factor as the “following guidelines” factor.

To investigate the generalizability of the relationship between perceived social and health impact and disease-relevant behaviors, we asked whether the observed perceptions were associated with behavior out-of-sample by using large-scale data from the Google Mobility Report^1^. The Google Mobility Report measures change in movement (i.e., number of visitors to or time spent in places of interest) from a baseline to a specific date. The baseline is the median movement measurement during a 5-week span before the COVID-19 pandemic, from January 3, 2020 to February 6, 2020. The places of interest included retail and recreation locations, grocery stores and pharmacies, parks, transit stations, workplaces, and residential locations. To match our individual impact perception data with state-level measurements of movement, we used the Google Mobility data for the state in which our study participants lived and on the date on which our study participants completed the questionnaire.

#### Analyses

The study was created and administered via Qualtrics Survey Software (Qualtrics, Provo, UT, United States). Analyses were conducted in R (version 3.6.3; R Core Team, 2019). We used the tidyverse package (Wickham et al., 2019) for data organization, lme4 package (Bates et al., 2014) for linear and mixed effects models, psych package for factor analyses and to generate composite factor scores (Revelle, 2020), and sjPlot (Lüdecke, 2021), and ggplot2 (Wickham, 2016) for visualization. In our linear mixed effects models, we center all variables on zero in accordance with recommended practices (Judd et al., 2009) and report specific coding for each variable in the results. The questionnaire items, de-identified data, and analysis code are available on Open Science Framework: https://osf.io/48yxk/. Because this investigation was exploratory, we used a predefined sample size of collecting at least 100 participants at each time point. Sample size was determined before any data analysis. A sensitivity analysis using G*Power (Faul et al., 2007) indicates that our final sample size of 188 would be sensitive to detect a correlation coefficient of *r* = 0.18 with 80% power.

### Results

#### Expected Severity of Social Impact Increases Over Time

We first examined participants’ overall expectations about the severity of the perceived health and social impact of the COVID-19 pandemic across time. In a linear mixed effects model, we regressed participants’ rating of perceived impact on domain (health = –0.5, social = 0.5), time frame (3 months from now = –0.5, 1 year from now = 0.5), and month of survey completion (April = –0.5, June = 0.5) with a by-participant random intercept and by-participant random slopes for domain and time frame. There was a main effect of domain, such that the anticipated health impact (*M* = 4.973) was rated as more severe than the anticipated social impact (*M* = 4.398; *b* = –0.566, *SE* = 0.108, *X*^2^(1) = 28.222, *p* < 0.001, 95% CI [–0.778, –0.354]). There was also a main effect of time, such that anticipated impact was rated as more severe for the next 3 months (*M* = 4.95) than 1 year in the future (*M* = 4.42; *b* = –0.520, *SE* = 0.085, *X*^2^(1) = 38.377, *p* < 0.001, 95% CI [–0.687, –0.354]). The main effect of month of survey completion was not significant (*M*_*April*_ = 4.64, *M*_*June*_ = 4.74, *b* = 0.098, *SE* = 0.183, *X*^2^(2) = 0.081, *p* = 0.776, 95% CI [–0.260, –0.456]).

**FIGURE 1:**
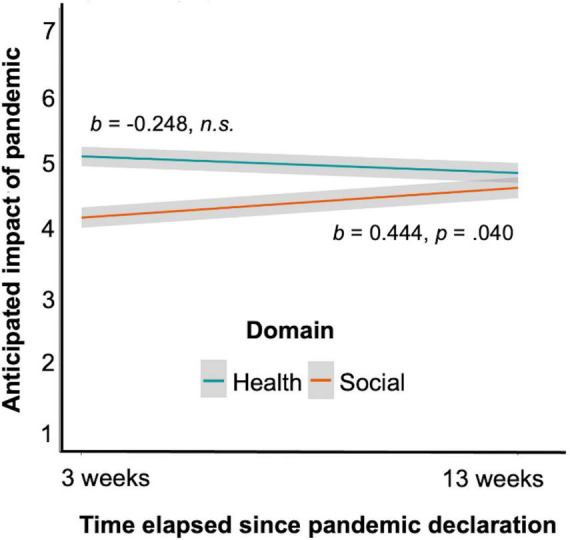
Ratings of perceived impact for each domain (health, social) on a scale of 1–7, by survey date. 3 weeks after the declaration of a global pandemic, participants rated the likely health impact of the pandemic as significantly more severe than the likely social impact. 13 weeks after the pandemic declaration, participants rated the likely health and social impacts as indistinguishably severe. Perceptions of the likely social (but not health) impact of the pandemic increased significantly across the two timepoints. Error bars represent standard error of the mean.

The main effects were qualified by three interactions, reflecting the interplay between perceptions across domains and time. First, there was an interaction between the time frame and the month of survey completion such that participants expected a greater difference in severity of impact between the next 3 months and 1 year in April as compared to June (*b* = 0.491, *SE* = 0.170, *X*^2^(1) = 8.327, *p* = 0.004, 95% CI [0.158, 0.824]). Second, there was an interaction between domain and time frame (*b* = 0.228, *SE* = 0.115, *X*^2^(1) = 4.028, *p* = 0.045, 95% CI [0.003, 0.453]); participants perceived a greater difference between the severity of health and social impacts within 3 months as compared to 1 year in the future. Finally, there was an interaction between domain and month of survey completion (*b* = 0.692, *SE* = 0.217, *X*^2^(1) = 10.207, *p* = 0.001, 95% CI [0.267, 1.116]; Figure 1). The difference in severity ratings between each health impact and social impact decreased from April to June. This change was driven by an increase in ratings of anticipated social impact (*b* = 0.444, *SE* = 0.216, *X*^2^(1) = 4.215, *p* = 0.040, 95% CI [0.020, 0.868]); ratings of anticipated health impact did not decrease (*b* = –0.248, *SE* = 0.208, *X*^2^(1) = 1.415, *p* = 0.234, 95% CI [–0.655, 0.160]). The three-way interaction between domain, time frame, and month of survey completion was not significant (*b* = –0.148, *SE* = 0.229, *X*^2^(1) = 0.418, *p* = 0.518, 95% CI [–0.597, 0.301]). Therefore, perceived impacts of the pandemic on the social domain appear to have increased over time and become more similar in magnitude to perceived health impacts (which did not change over time).

#### Expected Impact Relates to Likelihood of Engaging in Behaviors That Prevent Disease Spread

We examined whether perceived social and health impacts were associated with the intentional social interactions and following guidelines factor scores. Perceived health impact was positively associated with following guidelines (*r* = 0.342, *t*(184) = 4.943, *p* < 0.001, 95% CI [0.209, 0.463]), but not intentional social interactions (*r* = 0.049, *t*(184) = 0.662, *p* = 0.509, 95% CI [–0.096, 0.191]). Ratings of perceived social impact were positively correlated with social interactions (*r* = 0.311, *t*(184) = 4.431, *p* < 0.001, 95% CI [0.174, 0.435]; marginally correlated with following guidelines, *r* = 0.126, *t*(184) = 1.718, *p* = 0.088, 95% CI [–0.019, 0.265]). Therefore, perceived health impacts primarily related to following (non-social) guidelines, whereas perceived social impacts related to continued interaction with others.

**FIGURE 2:**
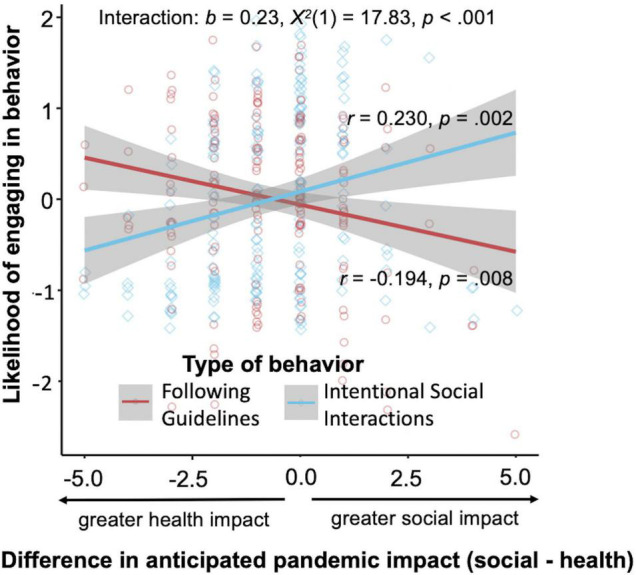
Association between relative expected impact of the pandemic in social vs. health domains and the likelihood of engaging in types of behaviors as defined by the *following guidelines* and *intentional social interactions* factor scores. As participants expected a higher social, as compared to health, impact, they reported that they would be more likely to interact with others and less likely to follow other (not interaction-related) recommended guidelines. Error bars represent standard error of the mean.

We observed considerable variability in severity of perceived impact, and a person’s behavior may track best with the *extent* to which anticipated social impacts are seen as *larger or smaller* than the anticipated health impacts in that person’s own mind. Additionally impact scores were positive correlated with each other (r = 0.36, *p* < 0.001). Therefore, we next calculated a difference score between ratings of social and health impact (social minus health). The difference score allows us to capture relationships with behavior given the extent to which people view the social or health impacts as larger. The social-health difference score was positively associated with intentional social interactions and negatively associated with following guidelines (intentional social interactions: *r* = 0.230, *t*(184) = 3.209, *p* = 0.002, 95% CI [0.089, 0.362]; follow guidelines: *r* = –0.194, *t*(184) = –2.682, *p* = 0.008, 95% CI [–0.329, –0.052]). The more highly participants rated the anticipated social, relative to health, impact, the more likely they were to report that they would interact with others and the less likely they were to report that they would follow recommended guidelines. We further confirmed that the social-health difference score was differently related to following guidelines vs. interacting with others by examining the interaction. We regressed the factor score ratings on the social-health difference score and type of behavior (following guidelines = –0.5, intentional social interactions = 0.5) with a by-participant random intercept. The interaction was significant (*b* = 0.233, *SE* = 0.055, *X*^2^(1) = 17.827, *p* < 0.001, 95% CI [0.125, 0.341]; Figure 2; main effects in the model were not significant, social-health difference score: *b* = 0.013, *SE* = 0.028, *X*^2^(1) = 0.213, *p* = 0.645, 95% CI [–0.042, 0.068], behavior: *b* = 0.144, *SE* = 0.101, *X*^2^(1) = 2.045, *p* = 0.153, 95% CI [–0.053, 0.342]), providing additional evidence that perceiving the social impact of the pandemic as relatively higher in severity is associated with less likelihood of following guidelines and greater likelihood of interacting with others.

##### Gender Effects

Because men and women may have responded differently to the pandemic (Carli, 2020), we sought to examine whether gender was associated with differences in behavior that tracked with perceived health and social impacts. Therefore, we included gender in the interaction term of the linear mixed effects model (women = –0.5, men = 0.5). There was a main effect of gender (*b* = 0.223, *X*^2^(1) = 4.799, *p* = 0.028, *95% CI* = [0.025, 0.422]), such that men provided higher ratings of impact overall. This was qualified by an interaction between gender and the factor score type (i.e., following guidelines, intentional social interactions), such that gender differences were larger for interacting with others (with men reporting they were more likely to interact with others) than for following guidelines (*b* = 0.575, *X*^2^(1) = 8.511, *p* = 0.004, 95% *CI* = [0.191, 0.959]). Additionally, the association between the social-health difference score and intentional social interactions was steeper for men than for women (*b* = 0.138, *X*^2^(1) = 6.146, *p* = 0.013, 95% *CI* = [0.029, 0.246]). That is, increases in perceived social (vs. health) impact were associated with greater likelihood of continued social interactions for men than for women.

#### Ratings of Impact Relate to State-Level Movement Change

To investigate the extent to which expectations about the health and social impacts of the pandemic were associated with behavior in everyday life, we capitalized on Google Mobility data, which aggregates location data collected unobtrusively from individuals’ mobile devices. Our primary measure was overall mobility, i.e., how far individuals traveled on a given day. In follow-up analyses, we additionally investigated *where* individuals tended to go. Existing research has documented state-level differences in health-related behaviors, e.g., mask-wearing (Fischer et al., 2021). We compared Mobility data from each of our participants’ state of residence on the date on which they completed our study to Mobility data from that same state on a control date, prior to the onset of the pandemic (see Procedures). We call this variable “movement change.”

There was no relationship between overall movement change and perceived health impact (*r* = –0.095, *t*(133) = –1.095, *p* = 0.275, 95% CI [–0.259, 0.076]). However, here was a significant correlation between overall movement change and perceived social impact. Specifically, in locations where our participants had reported anticipating higher social impact, there was less change (i.e., less reduction) in people’s mobility as reported by Google (*r* = 0.238, *t*(133) = 2.822, *p* = 0.006, 95% CI [0.072, 0.391]). In other words, those who resided in locations where people expected more social impact from the pandemic were less likely to curtail their social behavior. There was also a correlation with the social-health difference score, such that higher ratings of social vs. health impacts were associated with smaller changes in mobility (*r* = 0.301, *t*(133) = 3.640, *p* < 0.001, 95% CI [0.139, 0.448]).

**FIGURE 3:**
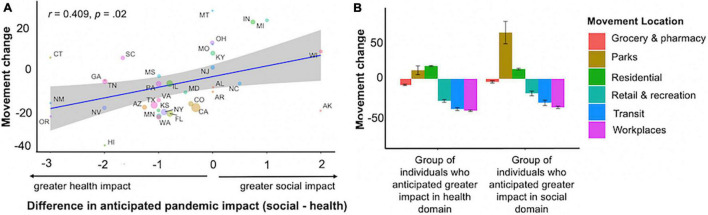
Relation between average individual ratings of expected impact by participants in a given geographic area (each state) and average movement change from baseline in that geographic area. Baseline was obtained from January 3, 2020-February 6, 2020 by taking the median value for each day. Error bars represent standard error of the mean. **(A)** People residing in states where health, relative to social, impact was estimated to be more severe were more likely to show less movement across the course of the pandemic, whereas people residing in states where social, relative to health, impact was estimated to be more severe were less likely to curb movement. **(B)** People who expected higher social impact spent more time in other locations and less time near home relative to those who expected a high health impact. For illustration, the graph depicts a group-based split of the data (higher expected impact for social domain group had social-health difference scores > 1, higher expected impact for health domain group had social-health difference scores < 1); the body of the manuscript reports correlations including the full range of difference scores.

Examining relationships with individual areas of movement (e.g., to grocery stores, parks), ratings of health impact were not correlated with movement change in any area. However, social impact was significantly positively related with movement in retail and recreation locations, grocery stores and pharmacies, parks, transit stations, and workplaces (all *p*s < 0.02; see Table 1 for full reporting of statistics). At the same time, there was a significant negative correlation between social impact and time spent in residential locations (*r* = –0.237, *t*(133) = –2.818, *p* = 0.006, 95% CI [–0.391, –0.071]). The associations with the social-health difference score corroborated these effects, with positive correlations with movement in all areas except residential locations, which had a negative correlation (all *p*s < 0.02; see Table 1 for full reporting of statistics; Figure 3).

**TABLE 1:** Correlations between in-sample perceptions of impact and out-of-sample state-level movement for individual locations assessed in the Google Mobility Report.

Health impact	*r*	*t*(133)	*p*	*95% CI*
Retail and recreation locations	–0.051	0.589	0.557	–0.218–0.119
Grocery stores and pharmacies	–0.014	–0.165	0.869	–0.183–0.155
Parks	–0.128	–1.492	0.138	–0.291–0.042
Transit stations	–0.028	–0.324	0.747	–0.196–0.142
Workplaces	–0.022	–0.256	0.798	–0.19–0.147
Residential locations	0.040	0.460	0.647	–0.13–0.207
**Social impact**				
Retail and recreation locations	0.201	2.365	**0.019**	0.033–0.358
Grocery stores and pharmacies	0.209	2.470	**0.015**	0.042–0.365
Parks	0.229	2.708	**0.008**	0.062–0.383
Transit stations	0.209	2.459	**0.015**	0.041–0.365
Workplaces	0.238	2.822	**0.005**	0.072–0.391
Residential locations	–0.237	–2.818	**0.006**	–0.391 – –0.071
**Social-health difference score**				
Retail and recreation locations	0.228	2.706	**0.008**	0.062–0.382
Grocery stores and pharmacies	0.203	2.393	**0.018**	0.035–0.360
Parks	0.323	3.936	**<0.001**	0.163–0.467
Transit stations	0.215	2.536	**0.012**	0.048–0.370
Workplaces	0.236	2.801	**0.006**	0.070–0.389
Residential locations	–0.251	–2.996	**0.003**	–0.403 – –0.086
**Social-health difference score 5 days later**				
Retail and recreation locations	0.219	2.588	**0.012**	0.052–0.374
Grocery stores and pharmacies	0.305	3.692	**<0.001**	0.0143–0.451
Parks	0.252	3.008	**0.003**	0.087–0.404
Transit stations	0.258	3.083	**0.002**	0.093–0.409
Workplaces	0.314	3.813	**<0.001**	0.153–0.459
Residential locations	–0.289	–3.479	**<0.001**	–0.436 – –0.126

*Bold values indicate significance at p < 0.05.*

We also tested whether the relationship between impact perceptions and movement hold when measured out to a future day, after the date on which participants completed our study. We assessed the relationship of perceptions measured during the survey to movement five days later. The higher participants rated the social vs. health impact of the pandemic, the less movement change (i.e., the less reduction in movement) was measured at the state level five days later (*r* = 0.285, *t*(133) = 3.431, *p* < 0.001, 95% CI [0.122, 0.433]). The relationship held for each of the location areas, and was again in the opposite direction for movement change in residential areas (*ps* < / = 0.01; Table 1).

### Interim Discussion

In the current study, we asked three main questions to understand the relationship between perceived social impact and disease-mitigating behavior: (1) whether there were differences across individuals and time in perceptions of the extent to which the pandemic would impact the health and social domains, (2) whether perceptions of impact were associated with likelihood of engaging in disease-preventing behaviors, and (3) whether ratings of impact generalized to a relationship with state-level movement change. We found that perceptions of the social impact of the pandemic increased between April and June (3 and 13 months after pandemic declaration, respectively). As a result, there was a smaller distinction in the anticipated severity of health vs. social impact at the second timepoint. Consistent with effects seen on social wellbeing (Chen et al., 2020), by 13 months into the pandemic, individuals expected a notable impact of the pandemic on our social lives, perhaps as a result of having experienced more cumulative direct social impacts of the pandemic (e.g., through missed holidays with family, sustained school closures). In findings bearing on our second question of interest, we replicated previous research indicating a relationship between perceptions of the health impact of the pandemic and self-reported engagement in recommended behaviors to mitigate the spread of the virus (Sheetal et al., 2020; Wise et al., 2020). We add to this research that perceptions of the social impact of the pandemic were associated with an increase in behaviors that have been shown to contribute to disease transmission, namely intentional interactions with others.

Finally, we observed an association between the perceptions of participants in our sample and the degree of movement change in their states. There were notable limitations with this approach, including that we only sampled a relatively small number of individuals across the United States and we did not measure additional demographic factors (e.g., socioeconomic status) that may interact with this effect. However, this finding points to the possible importance of local governance in promoting particular messages regarding the impact of the pandemic. We explored the possible influence of messages individuals might receive regarding the pandemic in Study 2.

## Study 2

In the first set of studies, we found evidence to support the idea that perceptions of the social and health impacts of the pandemic have different associations with the extent to which individuals are likely to follow disease-preventing recommendations. In a second study, we follow up on these findings with the aim of testing whether brief manipulations change individual perceptions of the social and health impacts of the pandemic. Health communication has been a key focus during the pandemic, particularly in light of evolving knowledge and information (Finset et al., 2020; Rains et al., 2020; Ratzan et al., 2020) and has the potential to change the perceptions that people hold about the possible health and social impacts. Yet, relatively little is known about how effective such messages might be. Additionally, in Study 2 we introduce imagined, hypothetical scenarios as a measure of behavioral intentions. In doing so, we can circumvent differences in the social opportunities that might be available to different people and ask all participants to consider how they would behave in the same scenarios. Finally, to follow up on the association between perceived impact and movement in locations measured in the Google Mobility report, we asked participants to consider the threshold of disease prevalence (as measured by number of cases) at which they would feel comfortable visiting each of the specific locations that were captured in the Google Mobility Report.

### Method

#### Participants

Participants were a sample of 362 adults recruited via Amazon’s Mechanical Turk between July 2, 2020 and July 6, 2020 (137 female, 224 male, 1 unknown; *age range* = 20–79 years, *M*_*age*_ = 38.65, *SD*_*age*_ = 11.73). All participants were living in the United States. For consistency with Study 1, we aimed to recruit 100 participants per group, however, forty-one additional participants were excluded for failing an attention check question that was repeated twice during the survey (participants were asked to write a specific phrase in a free response block to check that they were reading the instructions). Participants provided informed consent and received $0.75 for completing the task. The study was approved by the Institutional Review Board.

#### Design and Procedure

Participants were randomly assigned to conditions in a 2 × 2 design that varied based on the domain of focus (health, social) and severity of impact (high, low) creating four between-subjects conditions (high health *n* = 93, low health *n* = 84, high social *n* = 95, low social *n* = 90). In each of these conditions, participants saw a brief description of potential impacts of the pandemic. The impacts highlighted were taken from those that consistently loaded on the health and social factors in our survey (see Supplementary Materials for factor analysis details and survey available online for materials). We asked participants to imagine that they were living in a large city of approximately 500,000 residents and manipulated (within-subjects, counterbalanced) the case prevalence (high = participants read that the percent of cases has stayed over 10%, cases are increasing rapidly, and there have been 1,000 new cases in the past week; low = participants read that the percent of cases has stayed under 3%, cases are increasing slowly, and there have been 100 new cases in the past week). The first dependent variable was ratings of the likelihood of interacting in ten imagined scenarios that all involve potential interactions with other people (e.g., “Your neighborhood plans a small block party. Your elderly next-door neighbor will be there. How likely are you to go to the block party?”) on 5-point scale from 1 = not at all likely to 5 = extremely likely. For the second dependent variable, we asked participants to rate the highest number of new cases per week (continuous rating between 0 and 2000) at which they would feel comfortable engaging in various activities: going to work in an office building, going to the grocery store, going to a park, going shopping for clothes or other retail items, using public transportation, and going for a walk in their neighborhood. Participants also rated the perceived impact of the pandemic in health and social domains as in Study 1 (7-point scale from 1 = not at all severe to 7 = extremely severe).

### Results

#### Brief Descriptions Did Not Change Ratings of Impact

In order to examine whether brief descriptions influenced ratings of perceived impact, we regressed perceived impact on our manipulated degree of severity (low = –0.5, high = 0.5), order (low cases presented first = –0.5, high cases presented first = 0.5), and their interaction for those in the social condition. There was no effect of impact, order, or the interaction (*p*s > 0.50; see Table 2 for full reporting of model statistics). We conducted the same test for those in the health condition but regressed perceived health impact on the variables of interest. Again, none of the effects were significant (*p*s > 0.50). Therefore, it appeared that the brief manipulation was not sufficient to change perceptions.

**TABLE 2:** Effects of impact manipulation and presentation of prevalence order for each the social and health conditions of Study 2.

	Social condition				Health condition			
Predictors	*Estimates*	*CI*	*t-Value*	*p*	*Estimates*	*CI*	*t-Value*	*p*
(Intercept)	5.02	4.80–5.24	45.12	**<0.001**	5.09	4.88–5.29	48.69	**<0.001**
Impact	0.15	–0.29–0.59	0.67	0.506	0.01	–0.40–0.42	0.04	0.965
Prevalence order	–0.09	–0.52–0.35	–0.38	0.701	–0.12	–0.53–0.29	–0.58	0.561
Impact × prevalence order	–0.2	–1.08–0.68	–0.45	0.652	0.42	–0.40–1.25	1.01	0.313

*Bold values indicate significance at p < 0.05.*

#### Ratings of Perceived Impact Relate to Social Interactions in Imagined Scenarios

**FIGURE 4:**
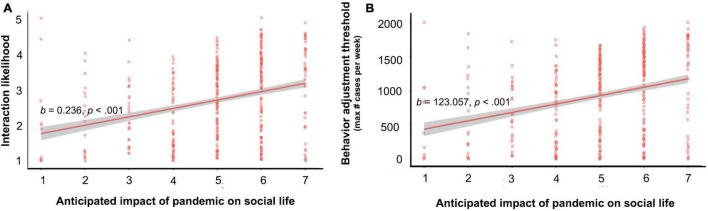
The relation between perceived social impact and intentional social interactions in hypothetical situations. **(A)** Participants’ self-reported likelihood of interaction (averaged across 10 imagined social scenarios). **(B)** The maximum number of new positive COVID-19 cases per week (in an imagined city of 500,000 residents) at which the participants self-reported remaining willing to visit various locations outside the home (averaged across six locations). Error bars represent standard error of the mean.

Because participants’ perceptions of health and social impact were robust to the descriptions provided, we collapsed across conditions to examine the effects of perceived social impact on behavior in imagined social scenarios. We ran a linear mixed effects model, regressing the interaction rating for each scenario on ratings of perceived social impact (mean-centered), and ratings of perceived health impact (mean-centered). We included random intercepts for participant, amount of cases, and the scenario rated. The main effect of social impact was significant (*b* = 0.246, *SE* = 0.043, *X*^2^(1) = 30.52, *p* < 0.001, 95% CI [0.153, 0.320]; Figure 4A) such that higher perceived social impact was associated with higher likelihood of interacting with others in the imagined scenarios (the effect of health consequences was not significant, *p* = 0.397).

#### Ratings of Perceived Impact Relate to Case-Level Thresholds for Engaging in Daily Activities

Next, we examined participants’ case-level thresholds for visiting the locations from the Google Mobility Report. We defined the threshold as the highest number of new cases per week at which they would feel comfortable doing an activity. There was a main effect of perceived social impact (*b* = 123.06, *SE* = 22.87, *X*^2^(1) = 28.96, *p* < 0.001, 95% CI [78.28, 167.84]; Figure 4B), suggesting that greater perceived social impact was associated with people continuing to visit the spaces with higher prevalence. The main effect of health impact was not significant (*p* = 0.98). The main effect of social impact held for work, grocery, parks, retail, transit, and residential locations (*p*s < 0.002; see Supplementary Table 12 for full reporting of model results by location).

### Interim Discussion

In Study 2 we first examined the effect of reading a brief description regarding the social or health severity of the pandemic on individual perceptions and found that participants’ perceptions were robust to this manipulation. Although these descriptions were not pre-rated by independent participants, their content was chosen based on the considerations that loaded on the social and health factors, respectively, in Study 1. At the very least, this suggests that simply articulating either the health or social impacts was insufficient to sway individuals’ perceptions. One possibility is that, given the salience of the pandemic at the time of data collection, it may have been difficult for participants to imagine they were in a different city with a particular case prevalence. Accordingly, we capitalized on individual differences in perceptions of the social impact of the pandemic to examine their effects on social interactions across imagined scenarios. We replicated the pattern in observed Study 1, such that higher perceived social impact was associated with participant report that they would be more likely to engage with others across the imagined scenarios.

Finally, participants considered the threshold of cases (in an imagined city) at which they would feel comfortable visiting the same places that were assessed in the Google Mobility Report. The findings of Study 2 supported our conclusions from Study 1: individuals who perceived a greater social impact of the pandemic reported that they would continue visiting public spaces, at a higher rate of case increase. These results may help us to understand, and potentially predict, how social interactions might change (or not) as cases climb. In conjunction with Study 1, we gain further confidence in the unique association between perceived social impact and engaging in social behaviors.

## Study 3

Given the consistency with which perceived health and social impacts were differentially associated with behavior, we were interested in the potential reasons *why* perceived social impact might be associated with social interactions. First, we focused on understanding the particular aspects of social life in which expected impact might relate to behavior. For example, people might be especially unwilling to reduce social interactions if they are concerned about the pandemic’s impact on their relationships or on their daily social interactions. Second, we investigated whether and how disease-mitigating social behaviors (e.g., reducing interactions with others) are seen as different from disease-mitigating physical behaviors (e.g., handwashing, wearing a mask). One possibility is that it is more difficult to curtail social behaviors (i.e., interacting with others) than it is to follow other guidelines, such as washing one’s hands more frequently. Another possibility is that one has less control over social behaviors than non-social behaviors. Alternatively, individuals may see social behaviors as overall less effective at reducing the spread of the disease.

### Method

#### Participants

Participants were 104 adults recruited via Amazon’s Mechanical Turk on May 7, 2020 (38 female, 63 male, 3 preferred not to respond; *age range* = 19–72 years, *M*_*age*_ = 40.13, *SD*_*age*_ = 13.07). All participants were living in the United States. For consistency with Study 1, we aimed to recruit 100 participants, but oversampled by 25 participants in order to account for potential exclusions. Twenty-one additional participants were excluded for using a limited number of response options, completing the survey in less than 5 min, and/or living outside of the United States. Participants provided informed consent and received $1.25 for completing the task. The study was approved by the Institutional Review Board.

#### Materials and Procedure

Participants rated the perceived impact of the pandemic in the next 3 months on 17 aspects of social life on a 7-point scale from 1 = large negative impact to 7 = large positive impact, with intervening values representing smaller impacts and the midpoint indicating “no impact.” Next, participants rated how likely they were to engage in each of 20 behaviors (10 each of social, e.g., interact in person with a stranger, and not social, e.g., wear a mask in a store) within the next month on a 5-point scale from 1 = not at all likely to 5 = extremely likely.

To assess whether social and physical behaviors were considered differently from each other, participants then rated how difficult it has been to do each of six behaviors in the last 30 days from 1 = very difficult to 5 = very easy (6 = NA, they don’t do this). The behaviors were: adjust the frequency of going to the store, practice recommended hand-washing practices, wear a mask in public, practice social distancing, stay away from close family and friends (with whom you don’t live), and avoid public spaces (behaviors chosen based on the intentional social interactions and following guidelines factors from Study 1). Participants rated the level of control they have in their ability to engage in these behaviors from 1 = almost none to 5 = a lot. Finally, they rated the behaviors in terms of effectiveness. Participants provided a numerical rating from 0 to 10 (0 = no impact at all, 10 = complete prevention) on the effectiveness of each of these behaviors for reducing the risk of COVID-19. They provided two effectiveness ratings: (i) effectiveness if they were to engage in the practices consistently themselves and (ii) effectiveness if their community in general were to engage in the practices consistently (We asked both questions because individuals may not believe that individual actions are sufficient to enact large-scale change in virus transmission but that such actions are effective when aggregated across individuals). Because individuals vary in their own risk of becoming seriously ill, we also included these ratings separately for the effectiveness in reducing the risk of COVID-19 for their self, close family and friends, people in their city, people in their state, and people across the globe. Finally, participants provided single-item ratings on how easy, controllable, and effective social practices (as a whole) and physical practices (as a whole) were, respectively, on a scale from 1 = not at all to 5 = very. Additional measures were collected that are outside the scope of this paper; these are provided in the survey available online.

#### Perceived Impact on Social Relationships Relates to Increased Social Interactions

Because participants only rated expected impact for areas of social life (as opposed to including areas that are not social in nature, as in Study 1), we tested whether these social variables would cluster in meaningful ways. The parallel analysis and scree plot recommended a three-factor solution, which accounted for 38% of the variance in people’s impact ratings. The first factor (15% of variance) captured impact on social contact between others (e.g., people’s ability to interact with acquaintances in person), the second factor (13% of variance) captured impact on social relationships (e.g., relationships between partners or spouses), and the third factor (10% of variance) captured global social issues (e.g., community cooperation). Again, for people’s likelihood of engaging in various behaviors, a three-factor solution was recommended (44% of variance), with the first factor representing social interactions (20%), the second factor representing following guidelines (15%), and the third factor containing a more heterogeneous group of variables (e.g., going to a park, 9%; see Supplementary Tables 13, 14 for factor loadings).

We examined whether perceptions of each of the three domains of social impact were associated with people’s likelihood of engaging in social interactions. Engagement in social interactions was positively associated with perceived impact on social relationships, consistent with the overall patterns observed in Studies 1 and 2 (*r* = 0.281, *t*(102) = 2.96, *p* = 0.004, 95% CI [0.094, 0.449]). However, engagement in social interactions was negatively associated with perceived impact on social contact and social interactions (*r* = –0.236, *t*(102) = –2.45, *p* = 0.016, 95% CI [–0.410, –0.045]), suggesting that the perceived impact of the pandemic on social *relationships*, as opposed to its effect on social contact more generally, best explained the likelihood of continuing to interact with others. The association between the global social issues factor score and social interactions was not significant (*r* = –0.023, *t*(102) = –0.234, *p* = 0.815, 95% CI [–0.215, 0.170]).

#### Social Behaviors Do Not Differ in Difficulty, Controllability, or Effectiveness From Other Guidelines

##### Difficulty

We regressed difficulty ratings on type of behavior (physical = –0.5, social = 0.5) with by-participant and by-activity random intercepts and a by-participant random slope. The effect of type of behavior was not significant (*b* = –0.148, *SE* = 0.222, *X*^2^(1) = 0.447, *p* = 0.504, 95% CI [–0.582, 0.286]). The effect of behavior type on the single-item measures asking how difficult it is to follow each physical guideline and social guideline was also not significant (*t*(193.99) = –1.102, *p* = 0.272, 95% CI [–0.437, 0.124]). That is, participants did not perceive adjustments to social behavior to be more difficult than following other guidelines. See Supplementary Table 15 for all pairwise comparisons, Bonferroni corrected.

##### Controllability

We ran the same tests as above to measure how controllable behaviors are. Again, there was no effect of type of behavior in the linear mixed effect model (*b* = –0.029, *SE* = 0.064, *X*^2^(1) = 0.210, *p* = 0.647, 95% CI [–0.155, 0.097]) or for the single item measure (*t*(198.57) = 0.539, *p* = 0.590, 95% CI [–0.188, 0.330]). None of the pairwise contrasts were significant (*p*s > 0.20; see Supplementary Table 16 for all pairwise comparisons).

##### Effectiveness

Finally, we looked at ratings for effectiveness, first examining ratings of effectiveness if the participant engaged in each behavior consistently. To test for differences in effectiveness, we regressed ratings of effectiveness on type of behavior (physical = –0.5, social = 0.5) with a random slope for type of behavior and random intercepts for participant, specific activity (e.g., handwashing), and target (e.g., self). The effect of type of behavior was not significant (*b* = 0.190, *SE* = 0.371, *X*^2^(1) = 0.263, *p* = 0.608, 95% CI [–0.565, 0.945]). The single-item comparison of effectiveness was also not significant (*t*(196.01) = –0.650, *p* = 0.517, 95% CI [–0.359, 0.181]). See Supplementary Table 17 for all pairwise comparisons. In sum, there was no difference in estimated effectiveness for social and physical behaviors.

Next, we ran the same model examining ratings of the effectiveness participants expected these behaviors to have if people in the community were to engage in them consistently. There was no effect of type of behavior on expected effectiveness (*b* = 0.277, *SE* = 0.390, *X*^2^(1) = 0.505, *p* = 0.477, 95% CI [–0.516, 1.070]). See Supplementary Table 17 for all pairwise comparisons. Taken together, recommendations related to social vs. health behaviors did not appear to differ in terms of difficulty, controllability, or expected effectiveness.

### Interim Discussion

In Study 3, we asked whether perceived impacts on specific aspects of people’s social lives were particularly influential in determining whether individuals would interact with others. Our results suggest that expected impacts on social relationships were especially meaningful in determining whether individuals would opt to participate in social interactions. Impact on one’s ability to engage in social contact did not increase self-reported interactions with others. These results may point to particular strategies for where to focus effects on reducing the social impacts of the pandemic (and therefore reducing social behavior that could contribute to virus spread). In Study 3, we also examined whether social behaviors were simply more difficult to enact or control or perceived as less effective than other physical-health promoting behaviors. Our results suggest that individuals do not view the social behaviors that could reduce disease spread as more difficult, less controllable, or less effective than their less social counterparts.

## General Discussion

Across three studies, we provide evidence that how individuals perceive the social impact of the pandemic is associated with the degree of continued interaction with others and engagement in disease-preventing behaviors. We provide support for these associations using participants’ self-reports of their own behavior, self-reports of behavioral intentions, and extrapolations to aggregated measures of movement occurring at the state level. Each of these approaches serves to help us understand these associations by probing individual self-reflection and intentions (via self-reported behaviors), reducing individual-level variability by having each participant consider a specific hypothetical situation (via imagined scenarios), and observing large-scale patterns in the real world (via state-level movement). Our third study highlights the pandemic’s impact on our social relationships as a potentially potent factor that influences individuals’ behaviors and decision making. These results expand upon previous research that implicates consideration of health effects (Sheetal et al., 2020; Wise et al., 2020) and individual characteristics (Alsan et al., 2020; Blagov, 2020; Bogg and Milad, 2020; Rothgerber et al., 2020) in the extent to which individuals follow disease-mitigating recommendations.

Further research is needed to better characterize the respective contributions to people’s behavior of their *relative* expectations of social vs. health impact, on the one hand, and their *absolute* expectations of social impact, on the other. Further understanding the relationship between expected health and social impacts could also highlight areas for intervention. Although Study 2 leaves open the question of exactly what type of intervention is needed to change individual perceptions about the pandemic, perceptions during the pandemic have changed (including social perceptions, Brañas-Garza et al., 2020; Van de Groep et al., 2020; Casoria et al., 2021). Additionally, changes in perceptions have been yoked to social distancing restrictions (Casoria et al., 2021) and health-relevant information including case prevalence and deaths (Brañas-Garza et al., 2020). Perceptions have been shown to be malleable and lead to meaningful change in other domains (e.g., intergroup relations; Pettigrew and Tropp, 2006), though evidence regarding the effectiveness of changing perceptions in health domains is less consistent (e.g., Elder et al., 1999; Goulding et al., 2010). More research is warranted to understand what type and level of intervention could directly (and causally) change individual perceptions about the impact of the pandemic and measure the relationship between changes in perceptions and changes in behavior.

The current studies have a collective strength of measuring the association between people’s expectations about the pandemic’s impact and people’s social behavior from multiple angles (e.g., self-report, state-level movement); however, it is worth noting that the interpretation of these findings is limited somewhat by their correlational nature. Although our findings are consistent with the possibility that expectations about how the pandemic will impact their social lives lead them to take measures to minimize those impacts (e.g., by continuing to interact socially), it may be also be the case that individuals who engage in more social interactions seek to justify that behavior by reporting elevated perceptions of social impact. On their own, the current studies cannot adjudicate between these possibilities (which are not mutually exclusive).

Although partly mitigated by the use of Google Mobility data, the conclusions that can be drawn from the present studies are additionally constrained by the reliance on self-report data. Responding in a questionnaire format may lessen some of the weight that people experience when making these decisions in real life. For example, it might be relatively easy to say that you would stay away from family and friends when completing the survey, but your choice may be different when faced with your own and others’ desire for social connection in the moment. Another notable limitation is that study measurement occurred amid the changing landscape of the pandemic. Across time when the studies were conducted, much changed (and continues to change) with regard to the number of cases, observed severity of impact on health and social consequences, and information provided to the public. Additionally, there is extensive variability in the extent to which individuals are impacted by the pandemic (e.g., Fortuna et al., 2020; Maroko et al., 2020; Benfer et al., 2021), and the populations most affected by the pandemic may not be well-represented in MTurk samples (Berinsky et al., 2012). We did not collect socio-economic information about our sample (but see Levay et al., 2016 for a description of general demographic characteristics of MTurk samples) and are therefore also restricted in our ability to relate individual level perceptions to state-level demographics. Therefore, there continues to be a high need for assessing how social impact—including perceptions of social impact—drive behavior across time and individuals during the ever-changing circumstances of the pandemic.

## Conclusion

The research presented here provides a first investigation of the role of perceptions of the impact of a pandemic on social life in influencing the extent to which individuals participate in disease-preventing behaviors, particularly with regard to social interactions. Expectations that the COVID-19 pandemic will more severely impact social life, and especially social relationships, are associated with greater engagement with others as the pandemic unfolds, which may hamper societal attempts to “flatten the curve” and accordingly prolong a pandemic. In this way, concerns that a pandemic will impact social life may eventually contribute to the very outcome people wish to prevent. These findings paint a more complete picture of how individuals weigh various aspects of a pandemic’s influence in their daily lives and provide some insights into the psychological mechanisms underlying, and therefore possible intervention targets for, behaviors that slow (or accelerate) the spread of communicable disease.

## Data Availability Statement

The datasets presented in this study can be found on Open Science Framework: https://osf.io/48yxk/.

## Ethics Statement

The studies involving human participants were reviewed and approved by University of Pennsylvania Institutional Review Board. The patients/participants provided their written informed consent to participate in this study.

## Author Contributions

RP collected the data and drafted the manuscript. AJ provided critical revisions. Both authors were involved with data analysis and conceptualized the research questions and contributed to the article and approved the submitted version.

## Conflict of Interest

The authors declare that the research was conducted in the absence of any commercial or financial relationships that could be construed as a potential conflict of interest.

## Publisher’s Note

All claims expressed in this article are solely those of the authors and do not necessarily represent those of their affiliated organizations, or those of the publisher, the editors and the reviewers. Any product that may be evaluated in this article, or claim that may be made by its manufacturer, is not guaranteed or endorsed by the publisher.

## Footnotes

1
https://www.google.com/covid19/mobility/
