# Supplementary material for: Anticipating Greater Impact of the COVID-19 Pandemic on Social Life Is Associated With Reduced Adherence to Disease-Mitigating Guidelines
Source: Front Psychol. 2022 Jan 27;12:756549. doi: 10.3389/fpsyg.2021.756549 (PMC8862145; doi:10.3389/fpsyg.2021.756549)
Supplement: Supplementary file 1 [file Data_Sheet_1.docx]

Supplemental Materials for:

*Anticipating greater impact of the COVID-19 pandemic on social life is associated with reduced adherence to disease-mitigating guidelines*

| Measures | Study 1 | Study 2 | Study 3 |
| --- | --- | --- | --- |
| Perceived Impact of the Pandemic | severity of consequences on health and social domains  (1-7 scale) | *Impact of the pandemic manipulated via vignettes across 4 between-subject conditions* | expected impact on 17 areas of **social** life (1-7 scale) |
|  | expected impact on 45 areas of life (1-7 scale) |  |  |
| Behavior | expected engagement in 26 behaviors (1-5 scale) | expected likelihood of interacting with others in 10 imagined scenarios | expected engagement in 20 behaviors (1-5 scale) |
| Movement | cell phone movement data matched to participants by state | threshold of cases at which participant reported feeling comfortable visiting locations |  |
| Additional |  |  | difficulty, controllability, and effectiveness of social and not social virus containment actions |

Table S1. Overview of measures collected and assessed across each study.

***Effects of Perceived Impact on Behavior Vary by Local Prevalence***

Because we were interested in how perceptions and behaviors related to the pandemic changed across time, we assessed whether the relationship between perceived severity of impact and behavior were influenced by the month of survey completion or local prevalence of cases. For these analyses, we excluded participants who reported that they did not know how many cases were in their city (n = 25) and, using a median split, separated those with 500 or fewer cases in their city (n = 82) and those with greater than 500 cases in their city (n = 81), regardless of total population. Using a linear model, we regressed the intentional social interactions factor score on the social-health difference score (mean-centered), local prevalence (fewer cases = -.5, more cases = .5), and the month of survey completion (April = -.5, June = .5). There was a main effect of the social-health difference score, such that higher ratings of anticipated social, relative to health, impact were associated with more intentional social interactions (*b* = 0.115, *SE* = 0.048, *t* = -2.424, *p* = .017, 95% CI [0.021, 0.209]). There was also a main effect of local disease prevalence, such that those living in a city with greater than 500 cases were more likely to interact with others (*b* = 0.418, *SE* = 0.150, *t* = 2.793, *p* = .006, 95% CI [0.122, 0.714]), likely reflecting a confound such that the locations with a higher case count are also likely to be more populous. All other effects were not significant (*ps* > .09).

When following guidelines was included as the dependent variable, there was only an interaction between prevalence and the social-health difference score (*b* = 0.249, *SE* = 0.099, *t* = -2.509, *p* = .013, 95% CI [0.053, 0.444]). For individuals who rated the severity of social impact as low relative to the severity of health impact, prevalence was not associated with the likelihood of following guidelines. However, those who rated social impact as higher than health impact reported that they would be less likely to follow guidelines when cases were fewer than 500.

Given the relative comparison of social to health impacts, a question arises as to the degree to which ratings of anticipated health *or* social impacts are primarily driving these effects. To better understand this possibility, we re-ran each model separately, controlling for ratings of health consequences and ratings of social impact, respectively. The effects above were robust to controlling for ratings of health impact (main effect of the social-health difference score on social interactions, *b* = 0.208, *SE* = 0.055, *t* = 3.796, *p* < .001, 95% CI [0.010, 0.316]; main effect of number of cases on social interactions, *b* = 0.352, *SE* = 0.147, *t* = 2.397, *p* = .018, 95% CI [0.062, 0.643]; interaction between prevalence and the social-health difference score on following guidelines, *b* = 0.233, *SE* = 0.096, *t* = 2.437, *p* = .016, 95% CI [0.044, 0.422]). However, when controlling for ratings of social impact, the main effect of the social-health difference score on intentional social interactions was not significant (*p* = 0.543; main effect of case prevalence remained significant, *p* = .018). Instead, there was a main effect of ratings of social impact, such that higher ratings were associated with higher likelihood of interacting with others (*b* = 0.176, *SE* = 0.056, *t* = 3.512, *p* = .002, 95% CI [0.065, 0.286]). Together, these findings suggest that the relationship between relative severity of impact and behavior was driven primarily by ratings of social impact (note: the converse model in which social ratings are included in the interaction term and the social-health difference score is included as a covariate still yields a main effect of social ratings). The interaction between local prevalence and the social-health difference score, described above, on following guidelines remained significant when we controlled for social ratings, and there was an additional main effect of social impact (*ps* < .05).

| Behavior | Intentional Social Interactions | Everyday Social Interactions | Following Guidelines |
| --- | --- | --- | --- |
| act outside of current guidelines in your location | 0.73 | 0.36 | -0.22 |
| adhere to current guidelines in your location | -0.2 | -0.02 | 0.72 |
| adjust walking or running route to maintain social distance | 0.1 | 0.09 | 0.53 |
| adjust the time you go to an essential business to reduce interactions | 0.06 | 0.09 | 0.59 |
| encourage others to self-quarantine | 0.17 | -0.06 | 0.64 |
| purchase extra groceries or supplied | 0.36 | 0.19 | 0.17 |
| donate to a global charity | 0.82 | 0.12 | 0.08 |
| go to the grocery store | 0.08 | 0.47 | 0.31 |
| practice recommended handwashing behavior | -0.33 | 0.03 | 0.56 |
| donate to a local charity | 0.74 | 0.22 | 0.13 |
| donate to a national charity | 0.82 | 0.14 | 0.1 |
| patron a local business | 0.4 | 0.55 | 0.16 |
| make an appointment for a routine doctor's visit | 0.73 | 0.21 | -0.07 |
| make an appointment for routine maintenance | 0.71 | 0.29 | -0.05 |
| self-quarantine if experiencing COVID-19 sx | -0.31 | 0.59 |  |
| get within 6 ft of an elderly individual | 0.61 | 0.41 | -0.14 |
| get within 6 ft of a family member | 0.58 | 0.54 | -0.13 |
| get within 6 ft of a neighbor | 0.64 | 0.43 | -0.16 |
| get within 6 ft of a stranger | 0.5 | 0.47 | -0.15 |
| interact in person (>6 ft) with an elderly person | 0.55 | 0.47 | -0.13 |
| interact in person (>6 ft) with a family member | 0.46 | 0.62 | -0.13 |
| interact in person (>6 ft) with a neighbor | 0.47 | 0.43 | 0.07 |
| interact in person (>6 ft) with a stranger | 0.38 | 0.63 | 0.03 |
| travel domestically | 0.78 | 0.23 | -0.11 |
| travel internationally | 0.74 | 0.14 | -0.16 |
| go outside for a walk | 0.04 | 0.39 | 0.27 |

Table S2. Factor loadings for self-reported behaviors across April and June in Study 1.

**“Everyday Social Interactions” Factor Score Results**

First, we examined the association between self-reported ratings of impact and the everyday social interactions factor score. The correlations with perceived social impact (*r* = 0.011, *t*(184) = 0.148, *p* = 0.992, 95% CI [-0.133, 0.155]) and perceived health impact (*r* = 0.114, *t*(184) = 1.550, *p* = 0.123, 95% CI [-0.031, 0.253]) were not significant. When we regressed the everyday social interactions factor score on the social-health difference score (mean-centered), local prevalence (fewer cases = -.5, more cases = .5), and the month of survey completion (April = -.5, June = .5), there was an interaction between the time of survey and the social-health difference score (*b* = 0.258, *SE* = 0.092, *t* = -2.823, *p* = .005, 95% CI [0.078, 0.439]; see Table S3 for full model results). Similar to the interaction with cases for following guidelines reported in the manuscript, there was no difference in likelihood to engage in everyday social interactions for those who rated health impact as relatively higher than social impact. However, for those who rated social impact as higher reported that they would be more likely to engage in everyday social interaction in June relative to those who completed the survey in April.


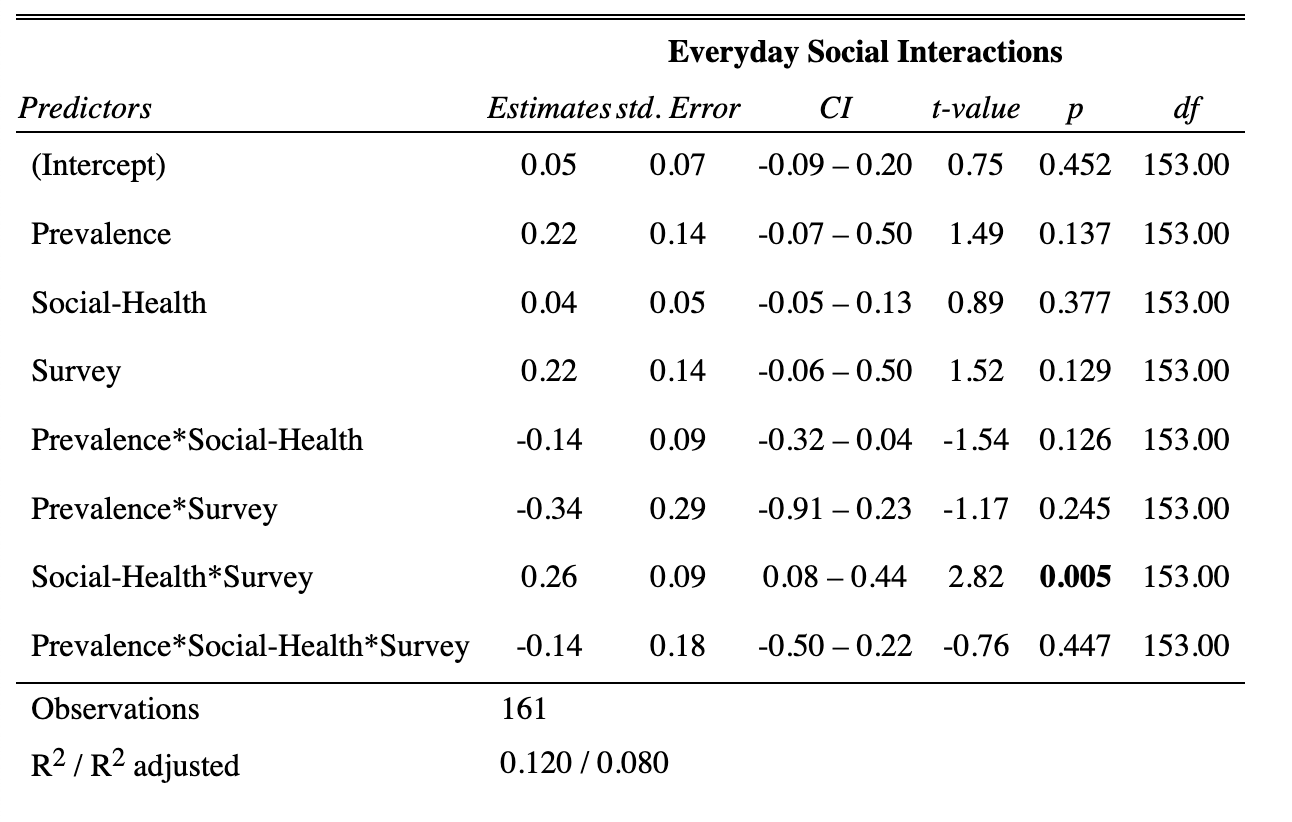


*Table S3.* Output from regression models examining the effects of local prevalence, the social-health difference score, and the month of survey completion on everyday social interactions.

**Associations with Behavior at Individual Time Points**

Because the distribution of cases across the country was different in April and June, we further explored the relationship between perceptions of severity of impact and behavior for each survey individually. Here we focused on whether factor scores, based on individual variables rated for their expected impact, would influence behavior. At each time point, participants rated the impact that they expected the pandemic to have in the next three months on 45 areas that spanned various domains, including health (e.g., survival rate of sick individuals, the nation’s healthcare system), social (e.g., ability to stay in touch with close family and friends, children’s social development), economic (e.g., employment, economic inequity), daily living (e.g., daily life routine and habits, ability to work remotely), and global (e.g., global violence, air pollution) areas in random order on a 7-point scale anchored by “1 = large negative impact” and “7 = large positive impact” with intervening ratings corresponding to smaller impact and the midpoint indicating “no impact.”

***Social Impact Distinguished from Other Types of Impact in April***

We ran a factor analysis on individual items that were rated in terms of expected severity of impact (using the absolute value of the impact rating). The parallel analysis and scree plot recommended a three-factor solution, which accounted for 35% of the variance. Variables relating to health impacts (e.g., hospitalizations, survival rate) tended to load on the first factor, accounting for 17% of the variance, variables relating to global impacts (e.g., pollution, education) tended to load on the second factor, accounting for 10% of the variance (though note that some social variables loaded heavily on this factor as well including impact on children’s social and emotional development), and variables relating to social impact (e.g., interactions with close family and friends, maintaining relationships) tended to load on the third factor, accounting for 8% of the variance (See Supplemental Materials Table S4 for factor loadings).

For the April survey alone, parallel analysis and the scree plot recommended a four-factor solution for the ratings of behaviors participants were likely to engage in. This solution explained 50% of the variance and the factors were: social interactions (16% of variance), following guidelines (8% of the variance), charitable acts (e.g., donating to charities, purchasing extra groceries to avoid the grocery store; 11% of the variance), and global interactions (e.g., travel, getting routine maintenance, 15% of the variance; See Supplemental Materials Table S5 for factor loadings).

| Area | Health | Global | Social |
| --- | --- | --- | --- |
| instances of domestic violence, child abuse and neglect | 0.27 | 0.33 |  |
| global air pollution | 0.38 |  |  |
| people's attitudes toward individuals from different nations | 0.24 | 0.66 |  |
| people's attitudes toward ingroup members | 0.33 | 0.51 |  |
| people's attitudes toward outgroup members | 0.62 |  |  |
| children's emotional development | 0.23 | 0.42 |  |
| children's social development | 0.26 | 0.5 |  |
| community cooperation | 0.6 |  |  |
| daily life routine and habits | 0.48 | 0.3 |  |
| economic inequity | 0.41 |  |  |
| educational outcomes for college students | 0.25 | 0.4 | 0.23 |
| educational outcomes for school-age children | 0.36 | 0.28 |  |
| people's employment | 0.75 | 0.31 |  |
| freedom of the press | 0.48 |  |  |
| frequency of illnes (not including COVID-19) | 0.41 |  |  |
| global coordination | 0.22 | 0.35 |  |
| global economy | 0.58 | 0.32 |  |
| global violence | 0.29 | 0.28 | 0.4 |
| engagement in health-related behaviors | 0.41 | 0.28 |  |
| global perception of your nation's healthcare system | 0.55 | 0.32 |  |
| your nation's healthcare system | 0.73 |  |  |
| hospitalizations among individuals | 0.63 | 0.27 |  |
| people's individual finances | 0.63 | 0.23 |  |
| people's ability to interact with close family and friends | 0.57 |  |  |
| acts of kindness by strangers | 0.37 |  |  |
| large industry | 0.35 | 0.31 | 0.39 |
| local economy | 0.6 | 0.38 |  |
| long-term health of individuals | 0.4 | 0.53 |  |
| people's ability to maintain relationships with close family and friends | 0.29 | 0.55 |  |
| people's ability to receive medical care for COVID-19 | 0.59 | 0.22 |  |
| people's ability to receive medical care for issues not related to COVID-19 | 0.57 | 0.32 |  |
| accuracy and completeness of media reports | 0.42 |  |  |
| mental health issues | 0.61 |  |  |
| migration of individuals | 0.27 | 0.37 |  |
| national economy | 0.7 | 0.26 |  |
| online learning | 0.38 | 0.48 |  |
| changes in people's political values | -0.2 | 0.53 |  |
| rate of poverty | 0.54 | 0.52 |  |
| small businesses | 0.64 | 0.33 |  |
| large-scale opportunities for social change | 0.2 | 0.32 |  |
| people's ability to stay in touch with close family and friends | 0.27 | 0.6 |  |
| survival rate of sick individuals | 0.51 | 0.39 |  |
| amount of individuals who experience COVID-19 sx | 0.6 |  |  |
| global water pollution | 0.41 |  |  |
| people's ability to work remotely | 0.25 | 0.29 |  |

Table S4. Factor loadings for self-reported impact for April in Study 1.

| Behavior | Time Frame | Global Interactions | Social Interactions | Charitable Acts | Follow Guidelines |
| --- | --- | --- | --- | --- | --- |
| act outside of current guidelines in your location | Next Week | 0.56 | 0.36 | 0.44 | -0.15 |
| adhere to current guidelines in your location | Next Week | -0.32 | -0.26 | -0.08 | 0.42 |
| adjust walking or running route to maintain social distance | Next Week | -0.04 | -0.08 | -0.02 | 0.55 |
| adjust the time you go to an essential business to reduce interactions | Next Week | -0.19 | -0.15 | 0.02 | 0.6 |
| encourage others to self-quarantine | Next Week | -0.33 | -0.08 | 0.28 | 0.51 |
| purchase extra groceries or supplied | Next Week | -0.08 | 0.14 | 0.46 | 0.2 |
| donate to a global charity | Next Week | 0.31 | 0.13 | 0.73 | 0.05 |
| go to the grocery store | Next Week | -0.04 | 0.2 | 0.2 | 0.49 |
| practice recommended handwashing behavior | Next Week | -0.64 | -0.2 | 0.09 | 0.36 |
| donate to a local charity | Next Week | 0.55 | 0.17 | 0.45 | 0.31 |
| donate to a national charity | Next Week | 0.46 | 0.11 | 0.63 | 0.17 |
| patron a local business | Next Week | 0.16 | 0.28 | 0.36 | 0.39 |
| make an appointment for a routine doctor's visit | Next Week | 0.53 | 0.01 | 0.47 | -0.09 |
| make an appointment for routine maintenance | Next Week | 0.58 | 0.17 | 0.44 | -0.1 |
| self-quarantine if experiencing COVID-19 sx | Next Week | -0.4 | -0.35 | -0.22 | 0.35 |
| get within 6 ft of an elderly individual | Next Week | 0.17 | 0.52 | 0.54 | -0.16 |
| get within 6 ft of a family member | Next Week | 0.23 | 0.57 | 0.51 | -0.06 |
| get within 6 ft of a neighbor | Next Week | 0.5 | 0.46 | 0.3 | -0.05 |
| get within 6 ft of a stranger | Next Week | 0.13 | 0.54 | 0.36 | -0.13 |
| interact in person (>6 ft) with an elderly person | Next Week | 0.4 | 0.42 | 0.3 | 0.03 |
| interact in person (>6 ft) with a family member | Next Week | 0.13 | 0.58 | 0.39 | -0.05 |
| interact in person (>6 ft) with a neighbor | Next Week | 0.19 | 0.37 | 0.32 | 0.14 |
| interact in person (>6 ft) with a stranger | Next Week | 0.18 | 0.53 | 0.3 | 0.18 |
| travel domestically | Next Week | 0.59 | 0.24 | 0.42 | -0.04 |
| travel internationally | Next Week | 0.64 | 0.13 | 0.42 | -0.1 |
| go outside for a walk | Next Week | 0.04 | 0.21 | 0.51 |  |
| act outside of current guidelines in your location | Next Month | 0.42 | 0.35 | 0.48 | -0.11 |
| adhere to current guidelines in your location | Next Month | -0.55 | -0.3 | -0.1 | 0.36 |
| adjust walking or running route to maintain social distance | Next Month | -0.17 | 0.12 | 0.05 | 0.55 |
| adjust the time you go to an essential business to reduce interactions | Next Month | -0.05 | -0.2 | 0.04 | 0.32 |
| encourage others to self-quarantine | Next Month | -0.1 | 0.18 | 0.37 |  |
| purchase extra groceries or supplied | Next Month | 0.08 | 0.11 | 0.59 | 0.04 |
| donate to a global charity | Next Month | 0.37 | 0.35 | 0.62 | -0.04 |
| go to the grocery store | Next Month | -0.06 | 0.15 | -0.28 | 0.55 |
| practice recommended handwashing behavior | Next Month | -0.72 | -0.18 | 0.09 | 0.24 |
| donate to a local charity | Next Month | 0.29 | 0.33 | 0.54 | 0.15 |
| donate to a national charity | Next Month | 0.57 | 0.2 | 0.48 | 0.11 |
| patron a local business | Next Month | 0.32 | 0.34 | 0.12 | 0.55 |
| make an appointment for a routine doctor's visit | Next Month | 0.48 | 0.1 | 0.23 |  |
| make an appointment for routine maintenance | Next Month | 0.62 | 0.34 | 0.24 | -0.06 |
| self-quarantine if experiencing COVID-19 sx | Next Month | -0.62 | -0.2 | -0.12 | 0.3 |
| get within 6 ft of an elderly individual | Next Month | 0.34 | 0.66 | 0.31 | -0.12 |
| get within 6 ft of a family member | Next Month | 0.06 | 0.75 | 0.26 | 0.05 |
| get within 6 ft of a neighbor | Next Month | 0.39 | 0.63 | 0.18 | -0.11 |
| get within 6 ft of a stranger | Next Month | 0.16 | 0.72 | 0.15 | -0.07 |
| interact in person (>6 ft) with an elderly person | Next Month | 0.28 | 0.69 | 0.12 |  |
| interact in person (>6 ft) with a family member | Next Month | 0.24 | 0.65 | 0.05 | 0.1 |
| interact in person (>6 ft) with a neighbor | Next Month | 0.29 | 0.6 | -0.03 | 0.21 |
| interact in person (>6 ft) with a stranger | Next Month | 0.4 | 0.59 | 0.04 | 0.05 |
| travel domestically | Next Month | 0.64 | 0.34 | 0.09 |  |
| travel internationally | Next Month | 0.77 | 0.29 | 0.13 | -0.06 |
| go outside for a walk | Next Month | -0.11 | 0.41 | -0.26 | 0.57 |

Table S5. Factor loadings for self-reported behaviors in April in Study 1.

***Ratings of Social Impact Interact and Local Prevalence Relate to Behavior in April***

We regressed the social interactions factor score on the social impact factor score and local prevalence (100 or fewer cases = -.5 (N = 36), >100 cases = .5 (N = 45)) and their interaction. The main effects of local prevalence (*b* = 0.371, *SE* = 0.202, *t* = 1.831, *p* = .071, 95% CI [-.032, 0.773]) and the social impact factor score (*b* = 0.071, *SE* = 0.125, *t* = 0.570, *p* = .570, 95% CI [-0.18, 0.319]) were not significant. The interaction was significant (*b* = -0.686, *SE* = 0.249, *t* = -2.753, *p* = .007, 95% CI [-1.182, -0.190]) such that, in locations with low local prevalence, higher ratings of social impact were associated with higher likelihood of social interactions (simple slope: *b* = 0.414, *SE* = 0.209, *t* = 1.982, *p* = .056, 95% CI [-0.010, 0.838]) whereas in locations with higher local prevalence, higher ratings of social impact were associated with marginally lower likelihood of social interactions (simple slope: *b* = -0.272, *SE* = 0.146, *t* = -1.859, *p* = .070, 95% CI [-0.567, 0.023]). Therefore, in April, individuals modulated their social behavior based both on their expectations of the social impact and the number of cases in their city. This relationship was present when examining the global impact factor as well (*b* = -0.499, *SE* = 0.242, *t* = -2.062, *p* = .043, 95% CI [-0.981, -0.017]). Again, the main effects were not significant: local prevalence: *b* = 0.314, *SE* = 0.212, *t* = 1.482, *p* = .142, 95% CI [-.108, 0.736], global impact factor score: *b* = 0.078, *SE* = 0.121, *t* = 0.646, *p* = .520, 95% CI [-0.163, 0.319]). In contrast, the relationship with the health impact factor score was a simple negative relation (*b* = -0.297, *SE* = 0.120, *t* = -2.473, *p* = .016, 95% CI [-0.537, -0.058]) such that higher ratings of health impacts were associated with lower social interactions. The effect of local prevalence (*b* = 0.163, *SE* = 0.217, *t* = 0.750, *p* = .455, 95% CI [-.270, 0.596]) and the interaction (*b* = -0.286, *SE* = 0.240, *t* = -1.191, *p* = .237, 95% CI [-.765, 0.192]) were not significant. Additionally, there were no effects of the social factor on the other behavior outcome factors (i.e., following guidelines, charitable acts, or global interactions; see Table S6 for full reporting of model results).


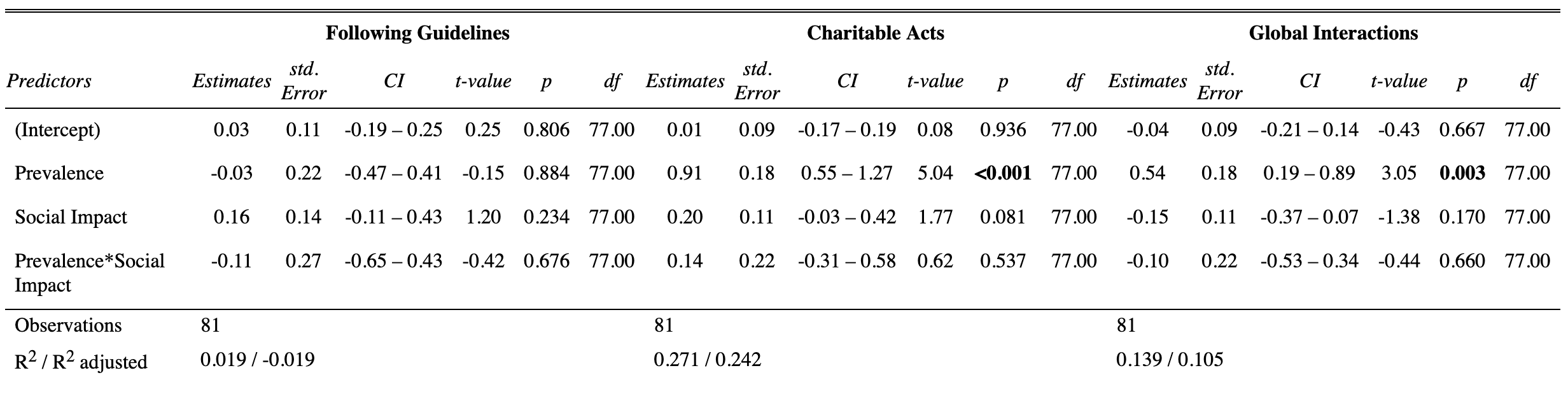


*Table S6.* Output from regression models examining the effects of local prevalence and perceived social impact on the following guidelines, charitable acts, and global interactions factor scores.

***Ratings of Social Impact and Local Prevalence Relate to State-Level Movement in April***

To test if ratings of impact projected to state-level movement measured by the Google Mobility Report we regressed movement on the number of positive cases (median split, obtained from The COVID Tracking Project (<https://covidtracking.com/>) for the date of survey completion). The interaction between prevalence and ratings of social impact emerged following a similar pattern as described above (*b* = -4.982, *SE* = 1.993, *t* = -2.500, *p* = .015, 95% CI [-8.950, -1.014]) such that, when cases were lower, higher ratings of social impact were associated with more movement (simple slope: *b* = 3.996, *SE* = 1.895, *t* = 2.109, *p* = .042, 95% CI [0.157, 7.836]). The direction of the relationship was negative when cases were high, but the simple slope did not reach significance (*b* = -0.985, *SE* = 0.897, *t* = -1.099, *p* = .279, 95% CI [-2.798, 0.828]). The main effect of the number of positive cases was also significant, indicating greater movement change (i.e., less movement; *b* = -10.591, *SE* = 1.625, *t* = -6.519, *p* < .001, 95% CI [-13.826, -7.356]; effect of social impact was not significant, *b* = 1.506, *SE* = 0.996, *t* = 1.511, *p* = .135, 95% CI [-0.478, 3.490]). When examining each of the locations separately, the interaction was significant for retail and recreation locations, grocery stores and pharmacies, and transit stations (*p*s < .01; Table S7). The interaction was not significant for parks or workplaces (*p*s > .1) and was significant in the opposite direction for residential locations (*b* = 1.944, *SE* = 0.796, *t* = 2.443, *p* = .017, 95% CI [0.360, 3.528]).


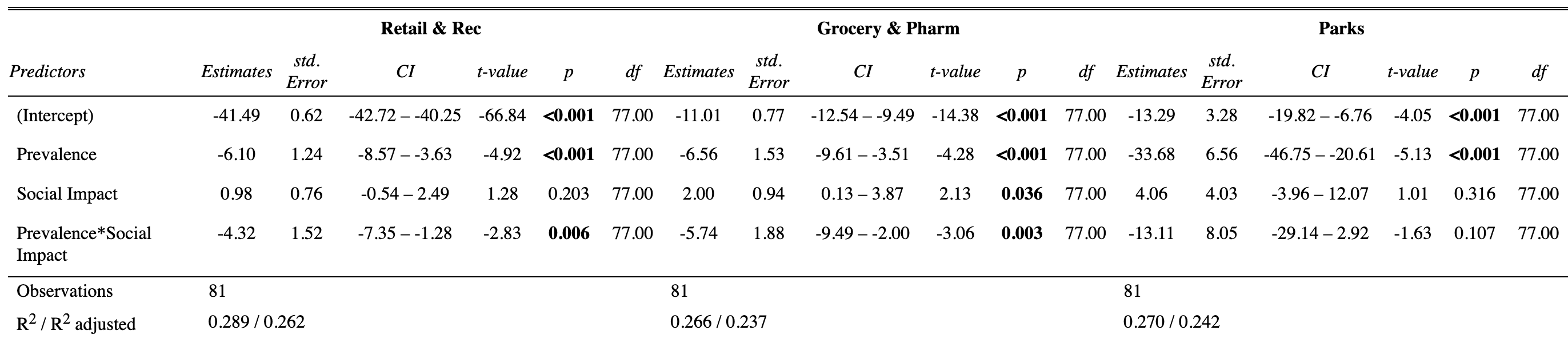


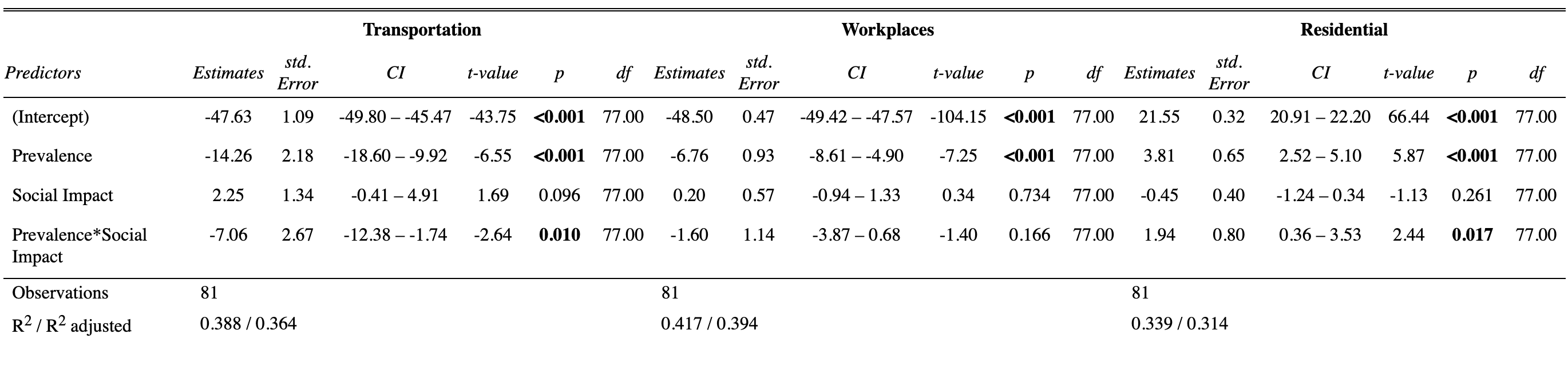


*Table S7.* Output from regression models examining the effects of local prevalence and perceived social impact on specific locations (i.e., retail and recreation, grocery and pharmacy, parks, transportation, workplaces, residential) assessed by the Google Mobility Report.

***Social Impact Less Distinguished from Other Types of Impact in June***

The parallel analysis and scree plot recommended a two-factor solution for ratings of impact, explaining 29% of the variance. While both factors contained some social variables, the first factor (15% of variance) was largely focused on health and economic impact and the second factor (14% of variance) was more centered on global and social impact. A three-factor solution was recommended for behavior, explaining 56% of the variance. Again, there was a factor related to social interactions (38% of variance) and a factor related to following guidelines (11% of variance). There was also a variable related to daily activities (e.g., going to the grocery store; 8% of variance; see Supplemental Materials Tables S8 and S9 for factor loadings).

| Area | Health | Social |
| --- | --- | --- |
| instances of domestic violence, child abuse and neglect | 0.47 | 0.22 |
| global air pollution | 0.3 | 0.43 |
| people's attitudes toward individuals from different nations | 0.56 |  |
| people's attitudes toward ingroup members | 0.62 |  |
| people's attitudes toward outgroup members | 0.2 | 0.45 |
| children's emotional development | 0.39 | 0.43 |
| children's social development | 0.32 | 0.4 |
| community cooperation | 0.63 |  |
| daily life routine and habits | 0.34 | 0.43 |
| economic inequity | 0.53 |  |
| educational outcomes for college students | 0.42 | 0.48 |
| educational outcomes for school-age children | 0.48 | 0.3 |
| people's employment | 0.66 |  |
| freedom of the press | 0.22 | 0.43 |
| frequency of illnes (not including COVID-19) | 0.32 | 0.36 |
| global coordination | 0.45 |  |
| global economy | 0.45 |  |
| global violence | 0.22 | 0.52 |
| engagement in health-related behaviors | 0.45 | 0.43 |
| global perception of your nation's healthcare system | 0.36 | 0.28 |
| your nation's healthcare system | 0.45 |  |
| hospitalizations among individuals | 0.54 | 0.24 |
| people's individual finances | 0.51 | 0.22 |
| people's ability to interact with close family and friends | 0.41 | 0.39 |
| acts of kindness by strangers | 0.6 |  |
| large industry | 0.42 | 0.24 |
| local economy | 0.66 |  |
| long-term health of individuals | 0.39 | 0.22 |
| people's ability to maintain relationships with close family and friends | 0.51 |  |
| people's ability to receive medical care for COVID-19 | 0.26 | 0.25 |
| people's ability to receive medical care for issues not related to COVID-19 | 0.33 | 0.36 |
| accuracy and completeness of media reports | 0.26 | 0.33 |
| mental health issues | 0.47 | 0.29 |
| migration of individuals | 0.41 |  |
| national economy | 0.56 |  |
| online learning | 0.28 | 0.35 |
| changes in people's political values | 0.33 |  |
| rate of poverty | 0.66 | 0.26 |
| small businesses | 0.63 |  |
| large-scale opportunities for social change | 0.22 | 0.61 |
| people's ability to stay in touch with close family and friends | 0.33 | 0.37 |
| survival rate of sick individuals | 0.38 | 0.22 |
| amount of individuals who experience COVID-19 sx | 0.35 |  |
| global water pollution | 0.51 |  |
| people's ability to work remotely | 0.35 | 0.46 |

Table S8. Factor loadings for self-reported impact for June in Study 1.

| Behavior | Social Interactions | Following Guidelines | Daily Activities |
| --- | --- | --- | --- |
| act outside of current guidelines in your location | 0.83 | -0.08 | 0.1 |
| adhere to current guidelines in your location | -0.15 | 0.87 | 0.23 |
| adjust walking or running route to maintain social distance | 0.15 | 0.64 | 0.08 |
| adjust the time you go to an essential business to reduce interactions | 0.19 | 0.53 | 0.07 |
| encourage others to self-quarantine | 0.25 | 0.65 | -0.27 |
| purchase extra groceries or supplied | 0.48 | 0.1 |  |
| donate to a global charity | 0.84 | 0.17 | -0.23 |
| go to the grocery store | 0.12 | 0.19 | 0.63 |
| practice recommended handwashing behavior | -0.29 | 0.44 | 0.28 |
| donate to a local charity | 0.81 | 0.22 | -0.05 |
| donate to a national charity | 0.81 | 0.17 | -0.12 |
| patron a local business | 0.54 | 0.13 | 0.47 |
| make an appointment for a routine doctor's visit | 0.83 | 0.08 |  |
| make an appointment for routine maintenance | 0.74 | 0.13 | 0.21 |
| self-quarantine if experiencing COVID-19 sx | -0.13 | 0.64 | 0.32 |
| get within 6 ft of an elderly individual | 0.75 | 0.11 |  |
| get within 6 ft of a family member | 0.72 | -0.03 | 0.25 |
| get within 6 ft of a neighbor | 0.78 | 0.01 | 0.23 |
| get within 6 ft of a stranger | 0.71 | -0.05 | 0.28 |
| interact in person (>6 ft) with an elderly person | 0.73 | -0.01 | 0.18 |
| interact in person (>6 ft) with a family member | 0.69 | -0.09 | 0.39 |
| interact in person (>6 ft) with a neighbor | 0.61 | 0.17 | 0.28 |
| interact in person (>6 ft) with a stranger | 0.54 | 0.1 | 0.45 |
| travel domestically | 0.81 | -0.02 |  |
| travel internationally | 0.76 | -0.04 | -0.09 |
| go outside for a walk | 0.08 | 0.15 | 0.61 |

Table S9. Factor loadings for self-reported behaviors in June in Study 1.

***Ratings of Severity of Social Impact Relate to Behavior in June***

In examining the relation between local prevalence (N = 44 “low” cases, N = 38 “high” cases; there were more cases overall in June, therefore low cases were defined as 1,000 cases or fewer) and ratings of social impact on behavior, the interaction was not significant in June (*b* = 0.060, *SE* = 0.228, *t* = 0.265, *p* = .792, 95% CI [-0.393, 0.514]; main effect of local prevalence was also not significant, *b* = 0.048, *SE* = 0.210, *t* = 0.228, *p* = .820, 95% CI [-0.370, 0.465]). However, the main effect social impact was significant (*b* = 0.389, *SE* = 0.114, *t* = 3.411, *p* = .001, 95% CI [0.162, 0.615]) such that higher ratings of impact were associated with higher likelihood of engaging in social interactions. When including the health factor in the model in place of the social factor, the relation was in the opposite direction (*b* = -0.387, *SE* = 0.117, *t* = -3.301, *p* = .001, 95% CI [-0.620, -0.154]; interaction was not significant, *b* = 0.220, *SE* = 0.234, *t* = 0.937, *p* = .352, 95% CI [-0.247, 0.687]; main effect of local prevalence was also not significant, *b* = 0.283, *SE* = 0.200, *t* = 1.421, *p* = .159, 95% CI [-0.114, 0.680]). When following guidelines replaced the dependent variable, the relations with the social factor and the health factor were both positive (social: *b* = 0.264, *SE* = 0.119, *t* = 2.212, *p* = .030, 95% CI [0.026, 0.502]; health: *b* = 0.250, *SE* = 0.128, *t* = 1.956, *p* = .054, 95% CI [-0.004, 0.504]). The interaction and main effects of local prevalence were not significant (interaction when social factor was the predictor, *b* = -0.001, *SE* = 0.239, *t* = -0.004, *p* = .997, 95% CI [-0.477, 0.475]; main effect of local prevalence when social factor was the predictor, *b* = 0.227, *SE* = 0.220, *t* = 1.034, *p* = .304, 95% CI [-0.210, 0.665]; interaction when health factor was the predictor, *b* = 0.157, *SE* = 0.255, *t* = 0.616, *p* = .540, 95% CI [-0.351, 0.665]; main effect of local prevalence when health factor was the predictor, *b* = 0.289, *SE* = 0.217, *t* = 1.330, *p* = .187, 95% CI [-0.143, 0.720]).

***Ratings of Social Impact and Local Prevalence Relate to State-Level Movement in June***

Because the number of cases was less positively skewed than in April, we regressed movement on the mean-centered number of cases and social impact (mean-centered). The interaction with cases was significant (*b* = -0.00004, *SE* = 0.00002, *t* = -1.974, *p* = .052, 95% CI [-0.00008, 0.0000006], while in opposite directions, neither of the simple slopes reached significance, low cases: *b* = 2.371, *SE* = 2.784, *p* = .400, high cases: *b* = -0.340, *SE* = 1.327, *p* = .799). There was also a main effect of number of cases (*b* = -0.00008, *SE* = 0.00002, *t* = -4.938, *p* < .001, 95% CI [-0.0001, 0.00005]; effect of social impact was not significant, *b* = -0.815, *SE* = 1.500, *t* = -0.544, *p* = .588, 95% CI [-3.794, 2.164]). The interaction was significant for retail and recreation locations (*p* < .02; Table S10), was marginal for grocery stores and pharmacies and parks (*ps* < .070), and was not significant for transit stations, workplaces, or residential locations (*p*s > .30).


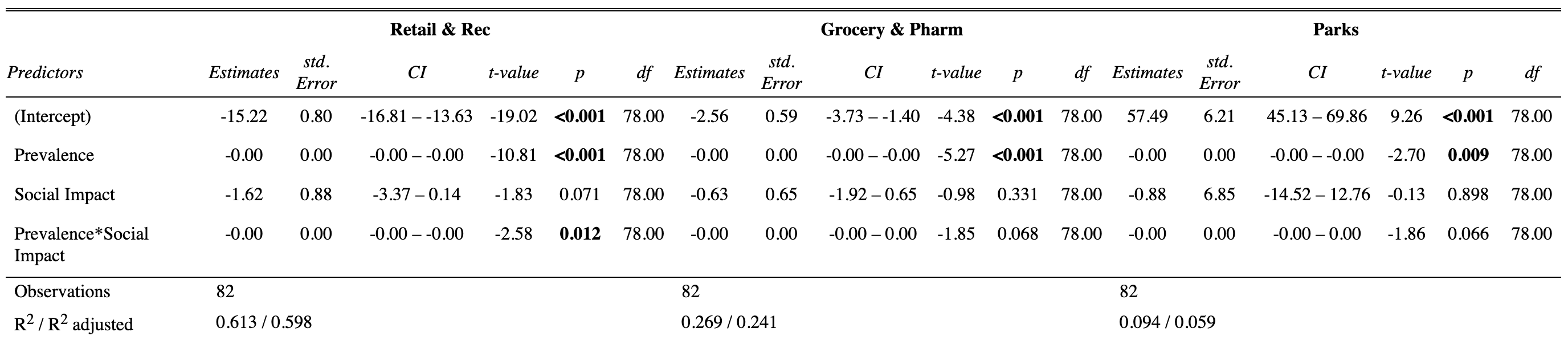


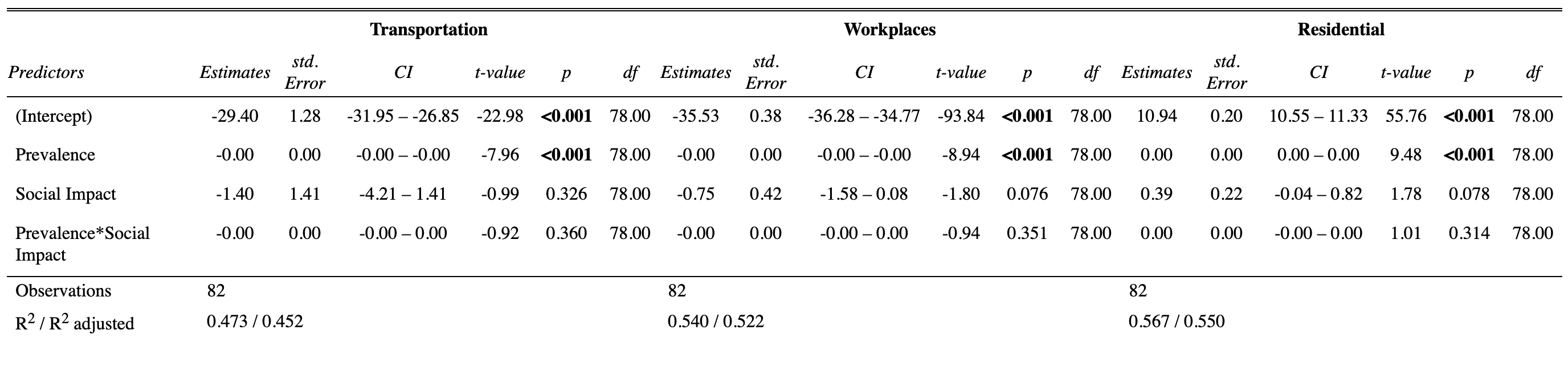


*Table S10.* Output from regression models examining the effects of local prevalence and perceived social impact on specific locations (i.e., retail and recreation, grocery and pharmacy, parks, transportation, workplaces, residential) assessed by the Google Mobility Report.


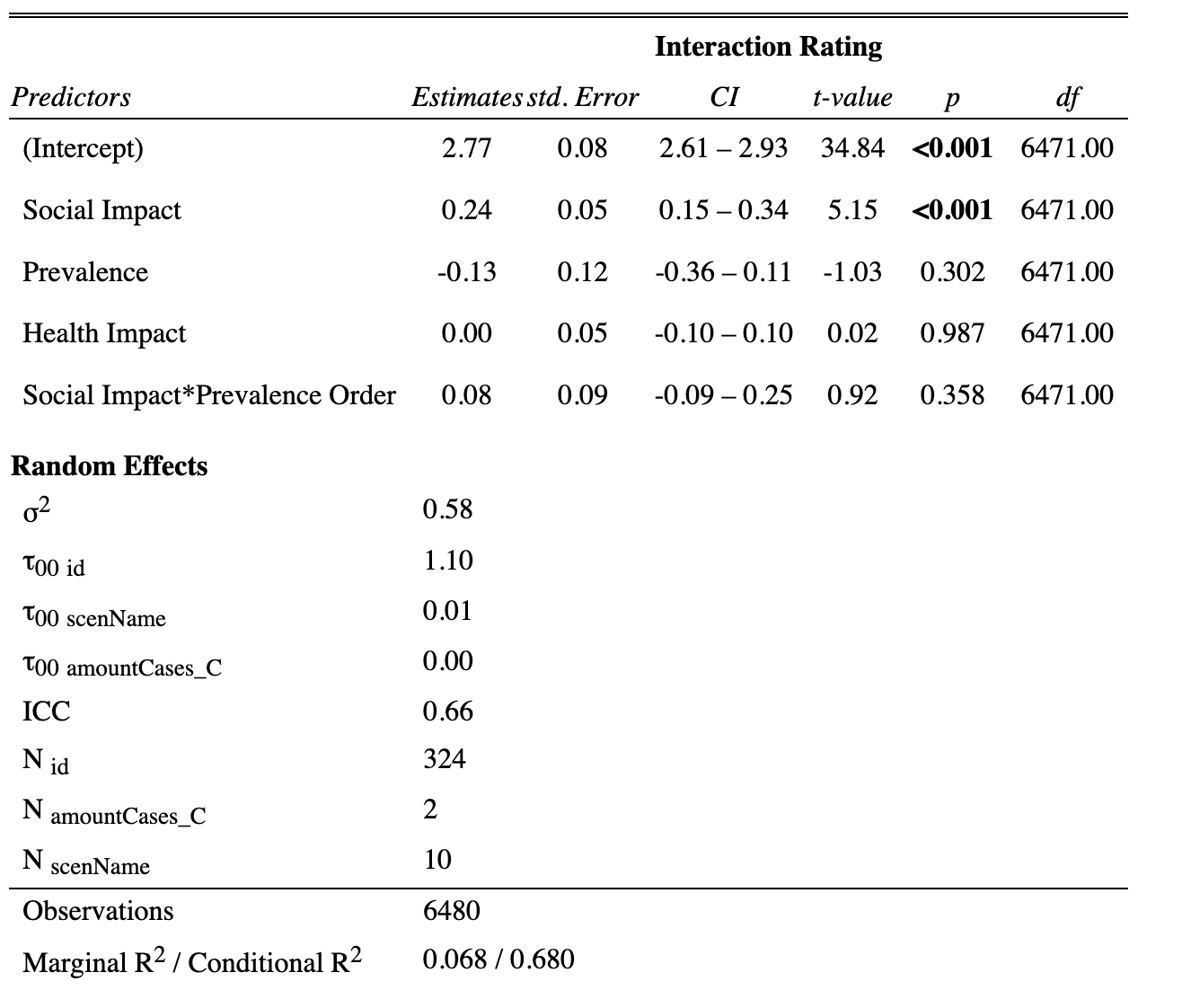


*Table S11.* Relationship between perceived impact and social interaction ratings for Study 2.


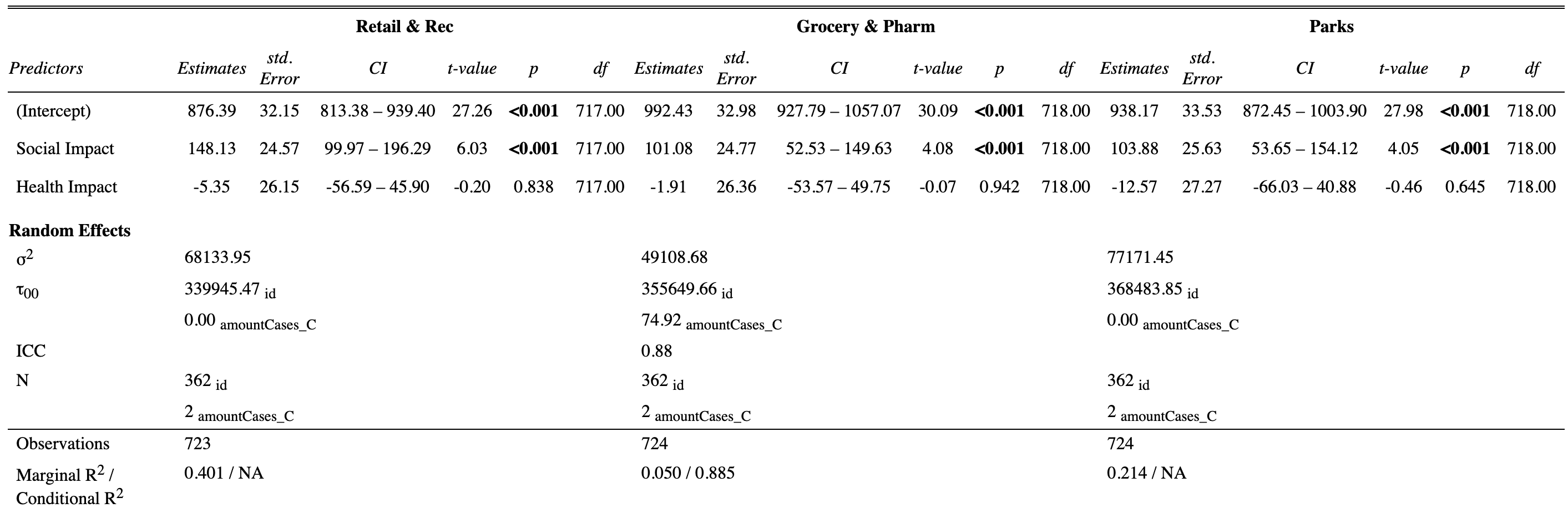


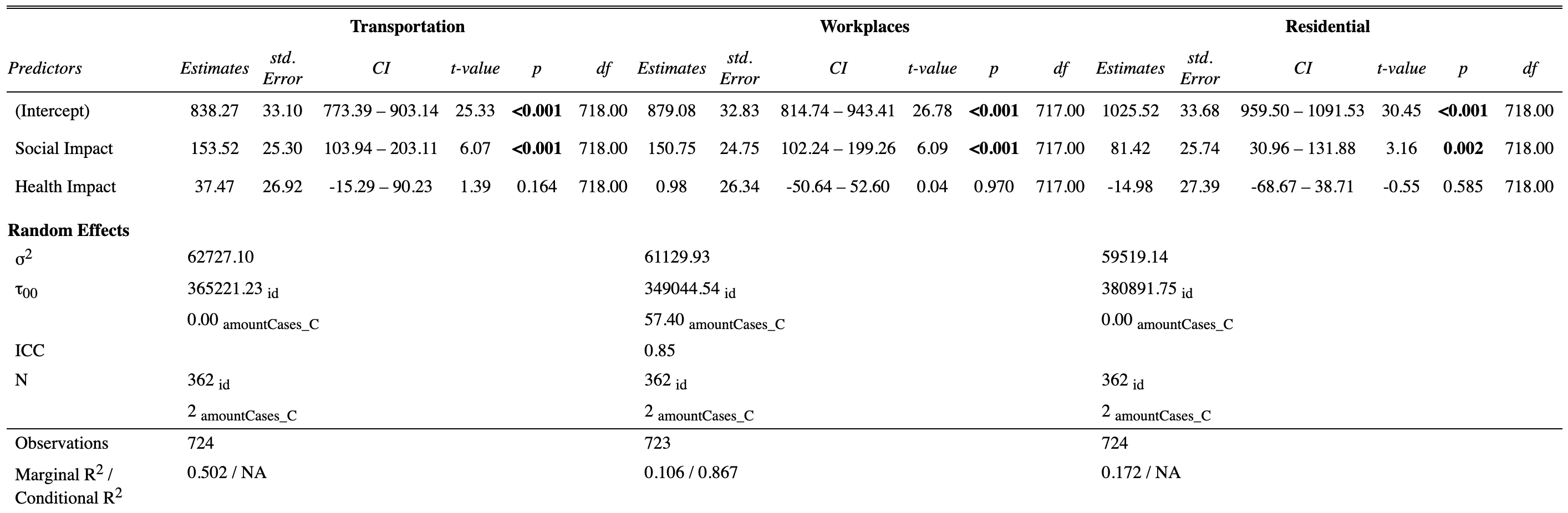


*Table S12.* Model effects for relation between social impact and maximum number of cases at which participants reported feeling comfortable interacting across individual location areas in Study 2.

| Area | Social Interactions | Social Relationships | Global Social Issues |
| --- | --- | --- | --- |
| people's attitudes toward ingroup members | 0.65 |  |  |
| community cooperation | 0.28 | 0.22 | 0.4 |
| people's ability to connect with new people | 0.33 | 0.49 |  |
| frequency of virtual social interactions | 0.55 | 0.4 |  |
| global coordination | 0.33 |  |  |
| people's ability to interact with acquaintances in person | 0.61 | 0.26 |  |
| people's ability to interact with their community in person | 0.65 |  |  |
| people's ability to interact with close family and friends in person | 0.56 |  |  |
| acts of kindness by strangers | 0.22 | 0.25 | 0.35 |
| people's ability to make new friends | 0.54 | 0.22 |  |
| online learning | 0.21 | 0.43 |  |
| parent-child relationships | 0.38 | 0.45 |  |
| people's ability to maintain relationships with acquiantainces | 0.27 | 0.53 |  |
| relationships between partners or spouses | 0.51 | 0.66 |  |
| people's ability to maintain relationships with close family and friends | 0.29 | 0.5 |  |
| opportunities for large-scale social change | 0.51 | 0.35 |  |
| people's ability to stay in touch with their community | 0.23 | 0.6 |  |

Table S13. Factor loadings for self-reported impact for Study 3.

| Behavior | Social Interactions | Following Guidelines | Miscellaneous |
| --- | --- | --- | --- |
| adhere to local guidelines | -0.3 | 0.56 | 0.36 |
| adjust your walking route to avoid other people | 0.53 |  |  |
| adjust the time you go to a store to avoid crowds | 0.57 |  |  |
| interact in person with a close family member or friend | 0.62 | 0.37 |  |
| interact in person with an elderly individual | 0.71 | 0.24 |  |
| go to the grocery store | 0.5 |  |  |
| practice recommended hand-washing practices | -0.31 | 0.42 | 0.45 |
| interact in person with a neighbor | 0.53 | 0.44 |  |
| interact in person with a stranger | 0.74 |  |  |
| wear a mask while walking outside | 0.71 |  |  |
| wear a mask in a store | 0.61 |  |  |
| go to a park | 0.41 | 0.49 |  |
| reach out in person to someone you don't know well | 0.73 |  |  |
| reach out in person to someone you care about | 0.42 | 0.24 | 0.4 |
| reach out virtually to someone you don't know well | 0.65 |  |  |
| reach out virtually to someone you care about | 0.33 | 0.57 |  |
| eat in a restaurant | 0.8 |  |  |
| self-quarantine | 0.73 |  |  |
| practice social distancing | -0.36 | 0.41 | 0.3 |
| get take-out or delivery | 0.25 | 0.23 |  |

Table S14. Factor loadings for self-reported behaviors in Study 3.

| *group1* | *group2* | *n* | *t* | *df* | *p* | *p.adj* |
| --- | --- | --- | --- | --- | --- | --- |
| adjust time you go to the store | avoid public places | 72 | -1.41 | 71 | 0.16 | 1 |
| adjust time you go to the store | handwashing | 72 | -3.38 | 71 | 0.001 | 0.02 |
| adjust time you go to the store | wearing a mask | 72 | -0.71 | 71 | 0.48 | 1 |
| adjust time you go to the store | social distancing | 72 | -1.69 | 71 | 0.10 | 1 |
| adjust time you go to the store | staying away from family and friends | 72 | 1.81 | 71 | 0.08 | 1 |
| avoid public places | handwashing | 72 | -1.87 | 71 | 0.07 | 0.99 |
| avoid public places | wearing a mask | 72 | 0.75 | 71 | 0.46 | 1 |
| avoid public places | social distancing | 72 | 0.19 | 71 | 0.85 | 1 |
| avoid public places | staying away from family and friends | 72 | 3.33 | 71 | 0.001 | 0.02 |
| handwashing | wearing a mask | 72 | 2.45 | 71 | 0.02 | 0.25 |
| handwashing | social distancing | 72 | 2.09 | 71 | 0.04 | 0.61 |
| handwashing | staying away from family and friends | 72 | 4.60 | 71 | 1.8E-05 | 0.0003 |
| social distancing | staying away from family and friends | 72 | 3.84 | 71 | 0.0003 | 0.004 |
| wearing a mask | social distancing | 72 | -0.54 | 71 | 0.59 | 1 |
| wearing a mask | staying away from family and friends | 72 | 2.33 | 71 | 0.02 | 0.34 |

*Table S15.* Pairwise comparisons for participant-rated difficulty in engaging in social and not social disease mitigation actions.

| *group1* | *group2* | *n* | *t* | *df* | *p* | *p.adj* |
| --- | --- | --- | --- | --- | --- | --- |
| adjust time you go to the store | avoid public places | 104 | 0.52 | 102 | 0.60 | 1 |
| adjust time you go to the store | handwashing | 104 | -0.27 | 102 | 0.79 | 1 |
| adjust time you go to the store | wearing a mask | 104 | 0.64 | 102 | 0.52 | 1 |
| adjust time you go to the store | social distancing | 104 | -0.18 | 102 | 0.86 | 1 |
| adjust time you go to the store | staying away from family and friends | 104 | 0.88 | 102 | 0.38 | 1 |
| avoid public places | handwashing | 104 | -0.80 | 102 | 0.42 | 1 |
| avoid public places | wearing a mask | 104 | 0.17 | 102 | 0.87 | 1 |
| avoid public places | social distancing | 104 | -0.82 | 102 | 0.41 | 1 |
| avoid public places | staying away from family and friends | 104 | 0.37 | 102 | 0.71 | 1 |
| handwashing | wearing a mask | 104 | 0.95 | 102 | 0.35 | 1 |
| handwashing | social distancing | 104 | 0.09 | 102 | 0.93 | 1 |
| handwashing | staying away from family and friends | 104 | 1.26 | 102 | 0.21 | 1 |
| social distancing | staying away from family and friends | 104 | -0.88 | 102 | 0.38 | 1 |
| wearing a mask | social distancing | 104 | 0.16 | 102 | 0.87 | 1 |
| wearing a mask | staying away from family and friends | 104 | 1.02 | 102 | 0.31 | 1 |

*Table S16.* Pairwise comparisons for participant-rated controllability of social and not social disease mitigation actions.

| *group1* | *group2* | *n* | *t* | *df* | *p* | *p.adj* |
| --- | --- | --- | --- | --- | --- | --- |
| **Effectiveness if Self Engaged in Behavior** | | | |  |  |  |
| adjust time you go to the store | avoid public places | 104 | -4.49 | 101 | 0.00 | 0.00 |
| adjust time you go to the store | handwashing | 104 | -4.69 | 101 | 0.00 | 0.00 |
| adjust time you go to the store | wearing a mask | 104 | -2.41 | 101 | 0.02 | 0.27 |
| adjust time you go to the store | social distancing | 104 | -4.71 | 101 | 0.00 | 0.00 |
| adjust time you go to the store | staying away from family and friends | 104 | -2.11 | 101 | 0.04 | 0.57 |
| avoid public places | handwashing | 104 | -0.96 | 101 | 0.34 | 1.00 |
| avoid public places | wearing a mask | 104 | 2.06 | 101 | 0.04 | 0.62 |
| avoid public places | social distancing | 104 | 0.06 | 101 | 0.95 | 1.00 |
| avoid public places | staying away from family and friends | 104 | 2.93 | 101 | 0.00 | 0.06 |
| handwashing | wearing a mask | 104 | 2.53 | 101 | 0.01 | 0.20 |
| handwashing | social distancing | 104 | 0.98 | 101 | 0.33 | 1.00 |
| handwashing | staying away from family and friends | 104 | 3.01 | 101 | 0.00 | 0.05 |
| social distancing | staying away from family and friends | 104 | -2.19 | 101 | 0.03 | 0.47 |
| wearing a mask | social distancing | 104 | 0.42 | 101 | 0.67 | 1.00 |
| wearing a mask | staying away from family and friends | 104 | 2.81 | 101 | 0.01 | 0.09 |
| **Effectiveness if Community Engaged in Behavior** | | | | |  |  |
| adjust time you go to the store | avoid public places | 104 | -4.99 | 100 | 0.00 | 0.00 |
| adjust time you go to the store | handwashing | 104 | -5.56 | 100 | 0.00 | 0.00 |
| adjust time you go to the store | wearing a mask | 104 | -2.56 | 100 | 0.01 | 0.18 |
| adjust time you go to the store | social distancing | 104 | -5.34 | 100 | 0.00 | 0.00 |
| adjust time you go to the store | staying away from family and friends | 104 | -3.52 | 100 | 0.00 | 0.01 |
| avoid public places | handwashing | 104 | -1.49 | 100 | 0.14 | 1.00 |
| avoid public places | wearing a mask | 104 | 2.99 | 100 | 0.00 | 0.05 |
| avoid public places | social distancing | 104 | -0.20 | 100 | 0.84 | 1.00 |
| avoid public places | staying away from family and friends | 104 | 1.82 | 100 | 0.07 | 1.00 |
| handwashing | wearing a mask | 104 | 3.52 | 100 | 0.00 | 0.01 |
| handwashing | social distancing | 104 | 1.21 | 100 | 0.23 | 1.00 |
| handwashing | staying away from family and friends | 104 | 2.52 | 100 | 0.01 | 0.20 |
| social distancing | staying away from family and friends | 104 | -3.71 | 100 | 0.00 | 0.01 |
| wearing a mask | social distancing | 104 | -1.00 | 100 | 0.32 | 1.00 |
| wearing a mask | staying away from family and friends | 104 | 2.08 | 100 | 0.04 | 0.60 |

*Table S17.* Pairwise comparisons for participant-rated effectiveness of social and not social disease mitigation actions based on whether the participant (self) or community engaged in the actions consistently.
